# Transcriptional regulation of the fidaxomicin gene cluster and cellular development in *Actinoplanes deccanensis* YP-1 by the pleiotropic regulator MtrA

**DOI:** 10.1128/spectrum.02702-23

**Published:** 2023-11-15

**Authors:** Huang Xie, Jing-Yi Ruan, Qing-Ting Bu, Yue-Ping Li, Yi-Ting Su, Qing-Wei Zhao, Yi-Ling Du, Yong-Quan Li

**Affiliations:** 1 First Affiliated Hospital and Institute of Pharmaceutical Biotechnology, Zhejiang University School of Medicine, Hangzhou, China; 2 Zhejiang Provincial Key Laboratory for Microbial Biochemistry and Metabolic Engineering, Institute of Pharmaceutical Biotechnology, Hangzhou, China; Shenzhen Bay Laboratory, Guangming, China

**Keywords:** fidaxomicin, protein-DNA interaction, two-component system, MtrA, transcriptome, morphological development, industrial actinomycetes

## Abstract

Fidaxomicin is an 18-membered glycosidic macrolide polyketide that was clinically used in the treatment of *Clostridium difficile* infection in 2011. Although the biosynthetic mechanism and pathway-specific regulator of fidaxomicin were well elaborated, little is known about the pleiotropic regulators outside the fidaxomicin gene cluster. To further reveal the transcriptional regulatory mechanism of fidaxomicin biosynthesis in *Actinoplanes deccanensis* YP-1, we used the 5′-biotin-labeled *fadR1* promoter (*fadS5R1p*) as a probe to affinity isolate the *fadS5R1p*-interactive protein MtrA, a response regulator of the two-component system MtrAB, from the proteome of mycelia. MtrA is bound directly to *fadS5R1p* to positively regulate gene cluster expression and fidaxomicin production. Fidaxomicin production was improved by 37% in the *mtrA* overexpressed strain. Furthermore, we revealed that MtrA tends to bind GTKAYS motifs and influence genes involved in secondary metabolites, phosphate transport, methionine transport, nitrogen metabolism, drug transport, membrane properties, etc. Overall, for the first time at the transcriptional level, we comprehensively revealed a pleiotropic regulator, MtrA, that not only directly mediates the FadR1-specific pathway to regulate the proper production of fidaxomicin but also participates in bacterial growth and development.

**IMPORTANCE:**

Cascade regulation networks are almost present in various kinds of microorganisms, but locating and systematically elucidating specific pleiotropic regulators related to a certain gene cluster can be a tricky problem. Here, based on the promoter of the fidaxomicin pathway-specific regulator FadR1, we utilized a “DNA to Proteins” affinity purification method and captured a global regulator MtrA, which positively regulates fidaxomicin biosynthesis. In the *mtrA* overexpressed strain, the production of fidaxomicin was improved by 37% compared to the native strain. Then, we combined the “Protein to DNAs” affinity purification method (DAP-seq) with the results of RNA-seq and systematically elucidated the primary and secondary metabolic processes in which MtrA directly or indirectly participates. Thus, our work brought up a new way to improve fidaxomicin production from the perspective of global regulation and analyzed the regulatory mechanism of MtrA. Meanwhile, we provided a novel methodology for the research of cascade regulation networks and vital secondary metabolites.

## INTRODUCTION

Actinomycetes from natural sources are widely recognized to produce a variety of antibiotics and other valuable secondary metabolites, including many antimicrobials, such as streptomycin, erythromycin, daptomycin, and FK506, with ingenious structures and potent biological activities (1, 2). The cascade regulatory network of these secondary metabolites is highly complex and vital for responding to changes in physiological and environmental conditions (3, 4). Two clades of intracellular regulators, including pathway-specific regulators and global/pleiotropic regulators, have been summarized in actinomycetes. The pathway-specific regulators are considered lower-level regulators, which are situated in the biosynthetic gene clusters (BGCs) of secondary metabolites and directly regulate the transcription of biosynthetic genes (5). In contrast to pathway-specific regulators, global/pleiotropic regulators are located outside BGCs and can not only affect the production of antibiotics but also regulate morphological differentiation and primary metabolism (6). Analysis of these regulators is of great importance to understand the molecular mechanisms of regulation, and it would be of great value in the pharmaceutical industry.

To date, a large number of pleiotropic regulators responsible for secondary metabolite production have already been identified. For instance, AtrA, a TetR family regulator, activates lidamycin production through binding to the promoter region of the *sgcR1R2* operon and activates the expression of SgcR1 and SgcR2, two of the three CSRs (cluster-situated regulators) within the lidamycin BGC (7). WblA, a WhiB-like protein first identified in *Streptomyces coelicolor*, functions as a global repressor of antibiotic biosynthesis, such as doxorubicin biosynthesis in *Streptomyces peucetius*, tautomycetin biosynthesis in *Streptomyces* sp. CK4412, and daptomycin biosynthesis in *Streptomyces roseosporus* (8 – 10). The master developmental repressor BldD acts as a key activator during daptomycin and avermectin production in *S. roseosporus* and *Streptomyces avermitilis* (11). In *S. coelicolor*, GlnR, a master nitrogen metabolism regulator, represses actinorhodin but activates undecylprodigiosin production directly via the pathway-specific activator genes actII-ORF4 and redZ (12). The response regulator MtrA was first characterized in the actinomycete *Mycobacterium tuberculosis* (MTB) and was proved to regulate jadomycin and chloramphenicol production in *Streptomyces venezuelae* (13).

Fidaxomicin (FDX), also known as tiacumicin B, lipiarmycin A3, clostomycin B1, PAR101, and difimicin, is a bacterial RNA polymerase inhibitor mostly used to treat *Clostridium difficile* infections (CDIs). Compared to vancomycin, FDX achieved significantly higher global cure rates and lower recurrence rates (14). Recently, the molecular basis of the narrow-spectrum activity of FDX on *Clostridium difficile* was established (15). Because of its clinical importance and market value, many efforts have been made to improve FDX production, among which transcriptional regulation plays an important role. Although we have characterized FadR1 as a LuxR-type pathway-specific activator, through which overexpression significantly increased FDX production in *Actinoplanes deccanensis* YP-1 (16), little is known about the pleiotropic regulators connecting to FDX production outside the FDX gene cluster.

In this work, to further reveal the transcriptional regulatory mechanism of FDX biosynthesis and improve FDX production, we used the 5′-biotin-labeled *fadR1* promoter (*fadS5R1p*) as a probe to directionally isolate the *fadS5R1p*-interactive protein MtrA, a response regulator of TCS MtrAB, from the proteome of *Actinoplanes deccanensis* YP-1. Further analysis revealed the regulatory mechanisms of MtrA, leading to its application in improving the yield of FDX and motif discovery.

## RESULTS

### Identification of MtrA as a *fadS5R1p*-interactive regulator through DNA affinity

**Fig 1:**
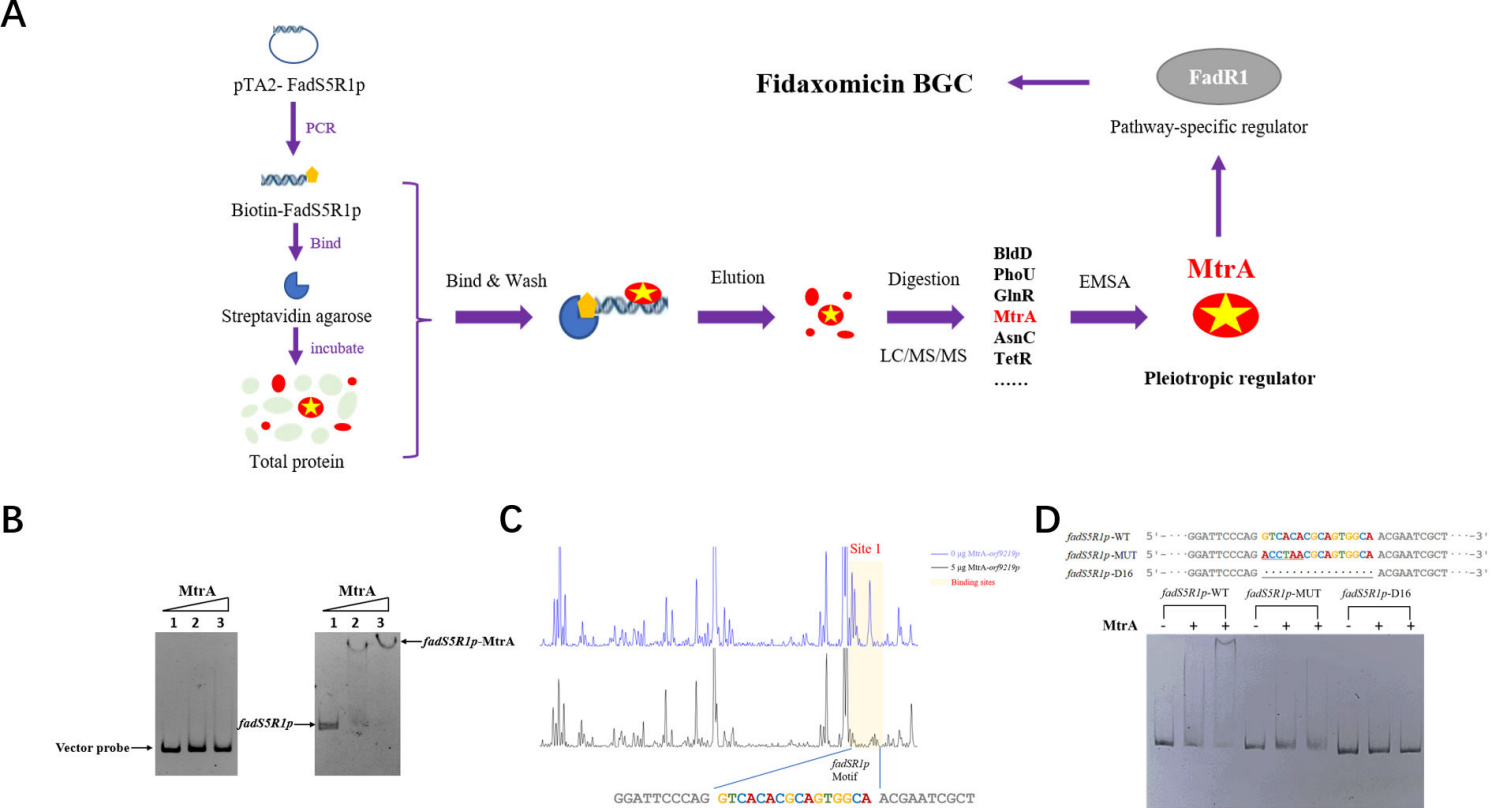
Identification of the *fadS5R1p*-interactive regulator MtrA. (**A**) Strategy for affinity isolation of *fadS5R1p*-interactive proteins from the *A. deccanensis* YP-1 proteome. (**B**) EMSA of MtrA binding to *fadS5R1p*. The 5-′fam-labeled void vector was the negative control. Lanes 1–3 are labeled fragments with 0, 0.05, and 0.1 µg of purified MtrA in a 10-µL mix. (**C**) DNase I footprinting assay. The blue chromatogram line is the group without MtrA, and the black chromatogram line is the group with the addition of 5 µg MtrA. The binding sites are covered with translucent orange rectangles. (**D**) EMSA with MtrA and mutant *fadS5R1p* probes. The underlined bases and ellipses indicate mutation and truncation sites of *fadS5R1p* probes. Reactions were carried out with the addition of no MtrA (lane 1) or with 0.025 µg (lane 2) or 0.05 µg (lane 3) MtrA in a 10-µL mix.

TiaR1 and TiaR2 were first found when the FDX gene cluster was initially sequenced in *Dactylosporangium aurantiacum* subsp. *hamdenensis* NRRL 18085 (17). We have reported that the FDX producer *Actinoplanes deccanensis* YP-1 contains the FDX BGC in high identity with that from *Dactylosporangium aurantiacum* subsp. *hamdenensis* NRRL 18085. FadR1, a LuxR-type transcriptional activator with 84.18% identity to TiaR1, binds to four promoter regions in the FDX gene cluster and greatly improves FDX production by overexpression. Regulator FadR1 and methyltransferase FadS5 are co-transcribed by the promoter *fadS5R1p* (16). To further reveal its transcriptional cascade-regulatory mechanism and improve fidaxomicin production, we used the biotinylated *fadS5R1p* DNA fragment as bait to isolate regulators outside the BGC that might interact with *fadS5R1p* directly from the total lysate of mycelia. The process of how we fished for specific regulators is shown in Fig. 1A. Altogether, 68 proteins (score > 20) were identified by LC/MS/MS (liquid chromatography-tandem mass spectrometry), and we picked up the top five regulator genes (*orf7349*, *orf1029*, *orf9379*, *orf9122,* and *orf9221*) according to the rank of coverage and peptides (Table S3).

Then, as shown in Fig. S1A, we used plasmid pET28a to express and purify Orf1029, Orf9379, Orf9122, and Orf9221 in *Escherichia coli* BL21 (DE3). The electrophoretic mobility shift assay (EMSA) results showed that Orf1029 (MtrA) specifically bound to *fadS5R1p* (Fig. 1B). Orf9379, Orf9122, and Orf9221 showed no signs of binding (Fig. S1B), while soluble Orf7349 cannot be obtained by far. At the same time, we overexpressed these regulators in *A. deccanensis* YP-1 and found that only the Orf1029 overexpressed strain showed significant FDX production improvement (Fig. S1C). Through NCBI BLAST analysis, we learned that Orf1029 (MtrA) is an OmpR family response regulator with a signal receiver domain and a helix-turn-helix DNA-binding domain, which is part of a two-component regulatory system named MtrAB. Here, we presented a DNase I footprinting assay for MtrA and *fadS5R1p*, and the results (Fig. 1C) showed that MtrA binds to *fadS5R1p* with a 16-bp (GTCACACGCAGTGGCA) area. In Fig. 1D, aimed at this binding area, we performed mutation and truncation experiments, and the mutant probes weakened or lost their abilities to be bound by MtrA. These results demonstrated that MtrA is a *fadS5R1p*-interactive regulator and activator of the fidaxomicin gene cluster.

### MtrA is required for *fadS5R1p* activity and FDX production

To further investigate the contribution of MtrA to fidaxomicin biosynthesis in *A. deccanensis* YP-1, *mtrA* was deleted from the YP-1 genome by the CRISPR/Cpf1 system (Fig. S2A). The disruption mutant Δ*mtrA* was confirmed by PCR analysis (Fig. S2B) and further verified by DNA sequencing (Fig. S2C). Meanwhile, the complementary strain Δ*mtrA::mtrA* was constructed by introducing Com-*mtrA*, containing *mtrA* and its own promoter, into the deleted mutant Δ*mtrA*.

**Fig 2:**
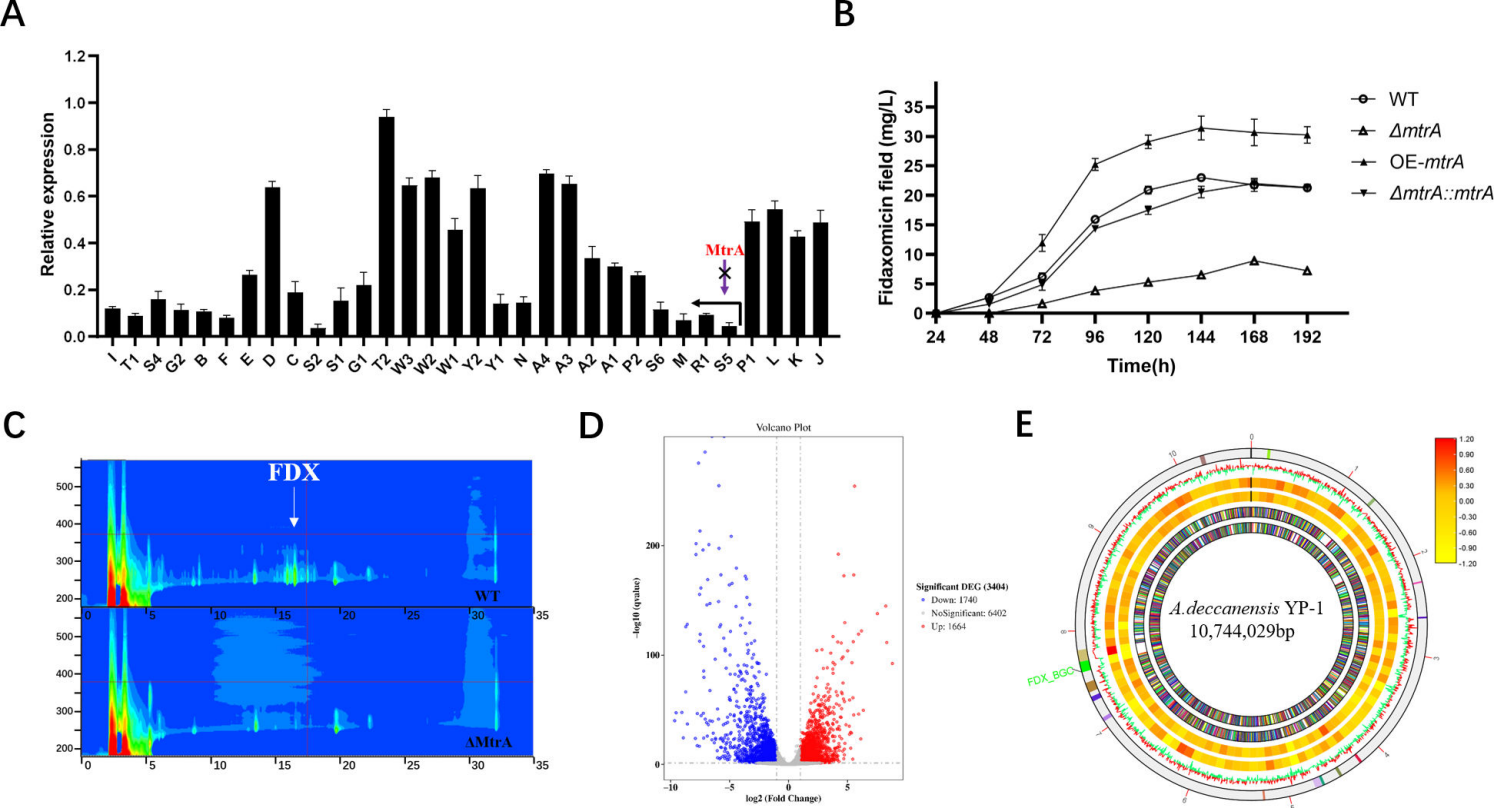
Schematic representation of the FDX biosynthesis, fermentation yield, and transcriptome analysis of Δ*mtrA*. (**A**) The effect of *mtrA* deletion on the transcription level of fidaxomicin cluster genes. The expression levels of fidaxomicin cluster genes are presented relative to the levels of corresponding genes in the wild-type (WT) sample, which were arbitrarily assigned a value of 1. The transcription level of hrdB was assayed as an internal control, and error bars were calculated by measuring the standard deviations among data from three replicates of each sample. (**B**) The effect of *mtrA* overexpression, deletion, and complement on the production of fidaxomicin. These strains were grown in YEME medium for 8 days, and fermentation broth was sampled at a 24-h interval. Vertical error bars correspond to the standard error of the mean of three replicated cultures. (**C**) HPLC analysis with a metabolic profile of fermentation broth in YEME between WT and Δ*mtrA*. (**D**) Volcano plot of regulated genes in Δ*mtrA* compared with WT. (**E**) Schematic representation of the distribution of BGCs and transcriptome patterns in the genome. From the outer circle to the inner circle, biosynthesis gene clusters (BGCs), 2d-log_2_FC of Δ*mtrA*-WT in line (red represents upregulated while green means downregulated), 2d-log_2_FC of Δ*mtrA*-WT in heat map, 4d-log_2_FC of Δ*mtrA*-WT in heat map, forward strand CDS, and reverse strand CDS are listed in sequence. The green part of the BGC circle represents the position of the FDX gene cluster.

To test how MtrA regulates FDX production by tuning the transcription levels of *fadR1*, *fadS5,* and associated downstream biosynthetic genes, we performed RT-qPCR (quantitative reverse transcription PCR) analysis. The transcription levels of FDX-associated biosynthetic genes were all decreased. Some genes directly controlled by the promoter *fadS5R1p* and regulator FadR1, like *fadS5*, *fadR1*, *fadM*, *fadS6*, *fadS2,* and *fadS1*, were at least reduced down to 20% relative expression in the *mtrA* deletion strain (Fig. 2A). At the same time, high-performance liquid chromatography (HPLC) analysis showed that deletion of *mtrA* greatly blocked fidaxomicin production by 61%, whereas FDX in the OE-*mtrA* strain improved by 37%, and reintroduction of *mtrA* basically rescued fidaxomicin production (Fig. 2B). The Δ*mtrA* strain presented a cleaner UV absorption spectrum, especially during the peak time of FDX (16.6 min) and its homologs (15.8, 16.1, and 17.4 min) compared to the wild type (WT) (Fig. 2C). Combined with the results of EMSA, it suggested that MtrA could bind directly to the *fadS5R1p* promoter and thus activate FadR1 expression to positively regulate fidaxomicin production.

Then, we performed comparative transcriptome analyses between the Δ*mtrA* and WT strains, and samples were taken at 48 and 96 h. Altogether, 9,806 genes were identified, among which 1,664 genes were upregulated and 1,740 genes were downregulated in the Δ*mtrA* mutant compared to the wild-type strain by the global analysis of differentially expressed genes (DEGs), as shown in Fig. 2D. The chromosomal distribution of DEGs between Δ*mtrA* and WT is shown in Fig. 2E. In the green segment of the BGC circle, which represents the distribution of the fidaxomicin gene cluster, we could see a significant downregulation of the FDX transcription level. All in all, we concluded that MtrA is essential for *fadS5R1p* activity and FDX production in *A. deccanensis*.

### Gene organization and function of *mtrAB-lpqB*

**Fig 3:**
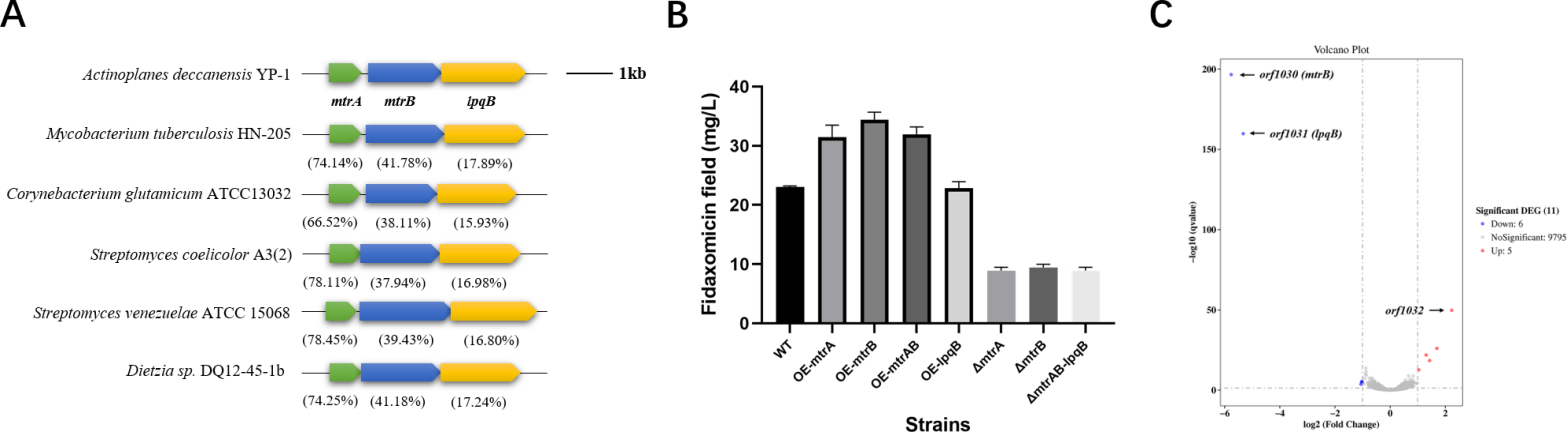
The relationships between *mtrA*, *mtrB,* and *lpqB*. (**A**) Genomic organization of the *mtrAB-lpqB* locus in *Actinoplanes deccanensis* YP-1 and other species. Orthologous genes are indicated by identical numbers or shading. Green: *mtrA*, response regulator MtrA; blue: *mtrB*, sensor kinase MtrB; golden: *lpqB*, lipoprotein LpqB. (**B**) FDX production influenced by overexpression and deletion of *mtrAB-lpqB*. (**C**) Volcano plot of regulated genes in Δ*mtrAB-lpqB* compared with ΔmtrA.

*mtrAB-lpqB*, sometimes called the three-component signal conduction system, performs in the same order as other actinomycetes, and the *mtrB* and *lpqB* genes are immediately downstream of *mtrA*. None of the surrounding genes are conserved so far. NCBI BLAST results revealed that these proteins are conserved in every actinobacterial genome sequenced to date, with the only exception of the obligate human pathogen *Tropheryma whipplei* (18). MtrA is the most conserved part among *mtrAB-lpqB*, and protein alignment showed that it had 74.14%, 66.52%, and 78.11% identity with its homologs from *M. tuberculosis*, *Corynebacterium glutamicum,* and *S. coelicolor* (Fig. 3A).

There is evidence that the essential response regulator MtrA in *M. tuberculosis* does not necessarily need its cognate sensor kinase MtrB for activation, which indicates that MtrA could be phosphorylated by crosstalk from a non-cognate sensor kinase or a small-molecule phosphordonor (18). Here, in *Actinoplanes deccanensis,* we separately knocked out *mtrA*, *mtrB,* and *mtrAB-lpqB* genes and respectively overexpressed *mtrA*, *mtrB*, *lpqB,* and *mtrAB* genes. Then, we got seven mutant strains (Δ*mtrA*, Δ*mtrB*, Δ*mtrAB-lpqB*, OE-*mtrA*, OE-*mtrB*, OE-*lpqB,* and OE-*mtrAB*). In Fig. 3B, we found almost the same reduced FDX production in the deletion strains, and FDX titers improved by about 50% in the OE-*mtrB* strain, higher than the OE-*mtrA* strain (37%). The OE-*lpqB* strain showed no significant changes. The fermentation results indicated that there is no non-cognate sensor kinase or response regulator of MtrA and MtrB in YP-1. Although the transcription level of *mtrB* and *lpqB* had not changed much after *mtrA* was deleted (Fig. S3), protein MtrB or LpqB can barely function in the absence of MtrA from the comparative transcriptomics results of Δ*mtrA* and Δ*mtrAB-lpqB* (Fig. 3C). Compared to approximately 400% FDX production improvement in the OE-*fadR1* strain, *mtrAB* overexpressed strains can just partially activate FDX production by no more than 50%. We suggested that the two-component system MtrAB is linked to a complicated regulatory network involving primary and secondary metabolism, and the inhibition effect of repressors, protein degradation, and the phosphorylation ratio of MtrA all contributed to the further improvement of FDX production. After all, it provided us with a new angle to activate or mute biosynthetic gene clusters by manipulating the expression of the MtrAB system.

### Deletion of MtrA affects morphological development and membrane properties

**Fig 4:**
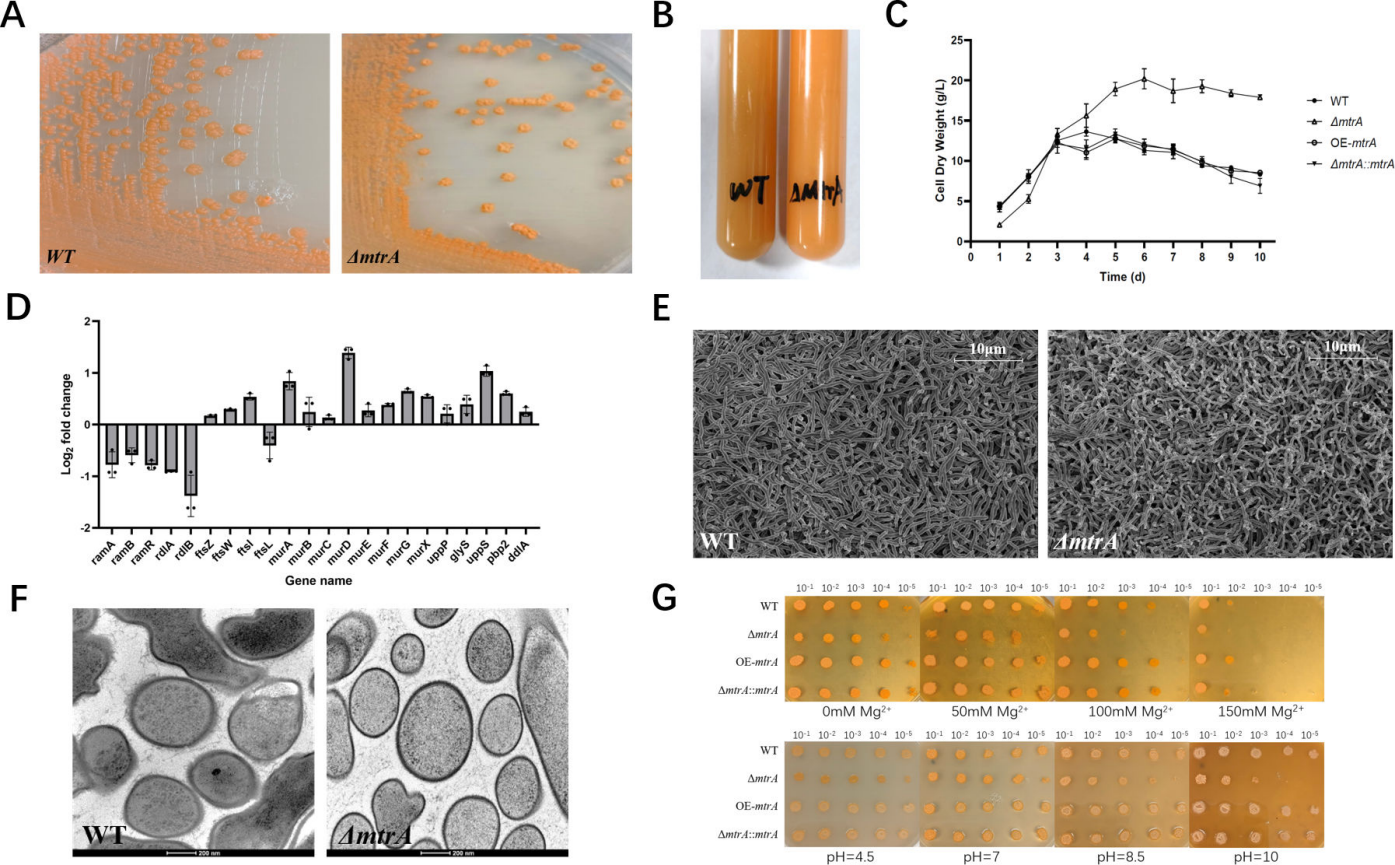
Influence of MtrA deletion on morphological development and membrane properties. (**A**) Phenotypes of WT and Δ*mtrA* grown on ISP4 medium for 6 days. (**B**) Phenotypes of WT and Δ*mtrA* grown on YEME medium (liquid transferred from flasks to tubes) for 7 days. (**C**) Growth curves of WT and Δ*mtrA* in fermentation experiments (*n* = 3, mean with SD). (**D**) Histogram of relative expression levels (log_2_FPKM*_ΔmtrA_*-log_2_FPKM_WT_) in DEGs involved in cell envelope homeostasis. (**E**) Scanning electron micrographs of the *Actinoplanes deccanensis* YP-1 wild type and the Δ*mtrA* mutant after cultivation for 2 days on ISP4 agar plates. The white bar represents a length of 10 µM. (**F**) Transmission electron microscopy (TEM) images of WT and Δ*mtrA* (white bar = 200 nM). (**G**) Comparison of osmotic pressure and alkali tolerance of WT, Δ*mtrA*, OE-*mtrA,* and Δ*mtrA::mtrA*. The first horizontal row indicates OD_600_ = 10^−1^, 10^−2^, 10^−3^, 10^−4^, and 10^−5^ (add 5 µL).

The knockout of *mtrA* caused global changes in *A. deccanensis* YP-1. Although both the wild-type and Δ*mtrA* strains do not produce spores, more stereoscopic and softer colonies are observed in the Δ*mtrA* strain (Fig. 4A), along with more orange pigment accumulation (Fig. 4B). The mutation not only severely hindered the production of FDX but also deteriorated early bacterial growth. Surprisingly, the Δ*mtrA* strain grew slower in the fore stage (≤48 h) but showed a much higher dry cell weight (DCW) during the anaphase of fermentation (Fig. 4C). Overexpression of *mtrA* did not have much influence on DCW but hindered the accumulation of orange pigment (Fig. S4A). In the conjugation transfer process, provided with the same concentration of donors and receptors, we found it hard to screen positive colonies for the Δ*mtrA* receptor (Fig. S4B). Meanwhile, we found that the mycelium of the Δ*mtrA* strain was much easier to resuspend after centrifugation (data not shown).

Thus, we thought there must be some morphological development and membrane-related genes influenced by MtrA. In Table S4, we found that some genes involved in the development of aerial hyphae (*ramABR* and *rdlAB*; downregulated) and peptidoglycan biosynthesis (*ftsZWIL*, *murABCDEFGX*, *uppP*, *glyS*, *uppS,* and *ddlA*; most upregulated) significantly changed in the Δ*mtrA* strain compared with the wild-type strain (Fig. 4D). These differentially expressed genes explained the phenomenon of delayed aerial hyphae formation in the beginning and rapid increasement in CDW (dry cell weight) afterward.

The scanning electron microscopy (SEM) results suggested that there are more irregular and narrow branches in the Δ*mtrA* mutant strain (Fig. 4E). In the view of ×15,000 magnification (Fig. S4C), we counted and calculated the mycelium diameter of WT and Δ*mtrA* strains, with a mean width of 424.26 ± 54.42 nm for the WT strain and 278.85 ± 41.99 nm for the Δ*mtrA* mutant (Fig. S4D). Transmission electron microscopy (TEM) images revealed that the outside part of Δ*mtrA* mycelia is smooth and without much “fluff.” Statistical analysis showed that the average cell envelope of Δ*mtrA* is slightly thinner than that of the wild type (Fig. S4E).

KEGG enrichment analysis showed that 92 genes of the “ABC transporters” pathway and 53 genes of the “Quorum sensing” pathway were greatly changed in the Δ*mtrA* mutant (Fig. S4F). To investigate whether MtrA of *A. deccanensis* YP-1 is involved in bacterial adaptation to environmental stresses, we compared the growth of the wild type and the Δ*mtrA* mutant under a variety of conditions causing cell envelope stress. As shown in Fig. 4F, the knockout of *mtrA* significantly suppressed the growth of mycelia under osmotic pressure and alkaline stress. The *mtrA* complementary strain and *mtrA* overexpressed strain exhibited similar growth to the wild-type strain under these culture conditions. Altogether, these findings indicated that MtrA plays a critical role in cellular morphological development and membrane properties.

### Identification of candidate genes and conserved motifs regulated by MtrA

**Fig 5:**
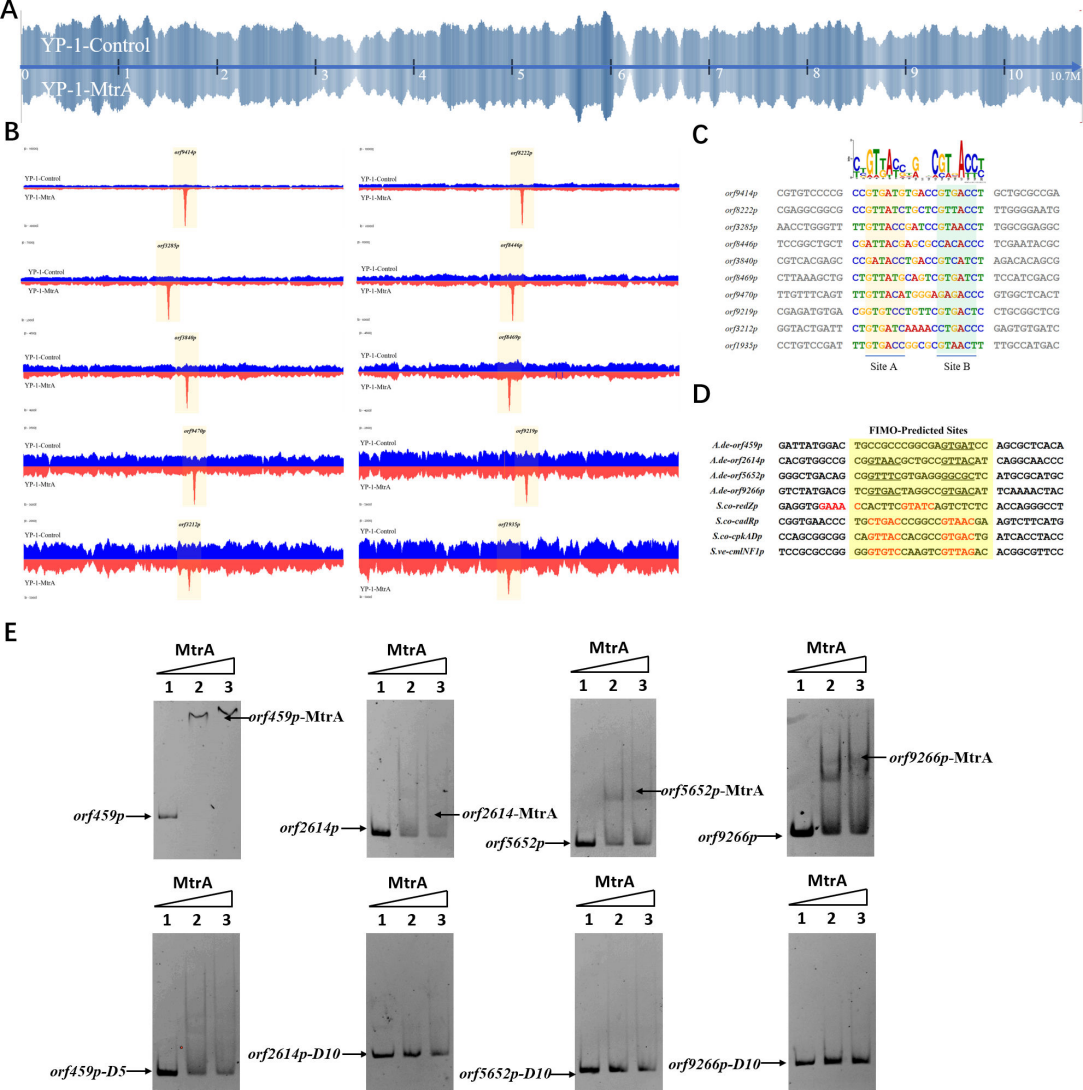
Identification of conserved motifs regulated by MtrA. (**A**) Genome-wide read coverage of YP-1-control and YP-1-MtrA based on DAP-seq results. (**B**) Top 10 significant MtrA binding areas on the whole genome of YP-1. (**C**) Predicted consensus MtrA motifs generated from MEME analysis based on the top 10 rough promoter areas. The *E*-value of the motif is 2.3e-009. The height of each nucleotide represents the relative frequency. (**D**) Predicted MtrA binding areas (covered with translucent yellow rectangles) generated from FIMO analysis based on the MtrA motif (experimental binding sites are marked in red font; underlined bases are predicted core sites to be deleted next). (**E**) EMSA of MtrA binding to *orf459p*, *orf2614p*, *orf5652p*, and *orf9266p* and their truncated probes. Lanes 1–3 are labeled fragments with 0, 0.05, and 0.1 µg of purified MtrA, respectively.

In order to find out the downstream genes directly regulated by MtrA, we mapped the genome-wide transcription factor-binding sites of the MtrA regulator by using DNA affinity purification sequencing (Fig. 5A). Combined with the transcriptome data of WT and Δ*mtrA*, we took the intersection of differentially transcribed genes (from RNA-seq results, |log_2_FC| ≥ 2) and genes controlled by characteristic promoters (from DAP-seq results, −log_10_(*P*-value) ≥ 5), and 75 genes were screened out (Table S5). Among these genes, the following are involved: 15 ABC transporters (transporting methionine, phosphate, oligopeptide, etc.), 10 regulators (including three phosphate regulatory proteins, two TCS-related proteins, one nitrogen regulatory protein, etc.), two drug transporter proteins (*orf459* and *orf5652*), two methyltransferases (*orf7032* and *orf9598*), one exodeoxyribonuclease III (*orf3212*), and one outer membrane stress sensor protease DegS (*orf9266*). When we focused on the BGC areas, it is noteworthy that only two genes, *fadR1* (*orf7031*) and *fadS5* (*orf7032*) located in the FDX BGC, were significantly found. The anti-SMASH results suggested that there are 16 BGCs located in 14 different parts of *A. deccanensis* YP-1, and we selected 16 core genes basically representing the level of BGC transcription (Table S4). Combined with the transcriptome results, we found that BGC-4, BGC-6, BGC-8, BGC-11, BGC-12-1, and BGC-12-2 are almost silent. BGC-2 (lanthipeptide, LAN) and BGC-13-1 (FDX) are significantly downregulated, while BGC-1 (OPT) is upregulated in the *mtrA* mutant strain (Fig. S5A). The EMSA results (Fig. S5B) indicated that MtrA did not participate in the direct regulation of OPT or LAN core genes (*orf161* and *orf1251*).

To investigate the binding sites of MtrA, we picked up the 10 most significant peaks in the promoter regions based on the DAP-seq results (Table 1). As shown in Fig. 5B, we visualized the sequencing data through Integrative Genomics Viewer (IGV) and localized 10 rough sequences (Table S6) accordingly. For detailed motif information, we uploaded these sequences to the MEME Suite web server and found an imperfect consensus binding motif (Fig. 5C, CYGTKAYSTGNBCGTGACCY) for MtrA. From the motif logo, it is easy to find out two sites A and B containing short GTKAYS-rich motifs and separated by 5 nt.

**TABLE 1:** 10 most significant peaks in the promoter regions based on DAP-seq^*a*^

Gene ID	Description	2-day log_2_FC	−log_10_ *P*-value
***orf9414***	Oligopeptide ABC transporter, periplasmic oligopeptide-binding protein OppA	−2.77	3,282.65
***orf8222***	PQQ-binding-like beta-propeller repeat protein	2.43	2,077.99
***orf3285***	Multimodular transpeptidase-transglycosylase	−5.66	1,224.43
*orf3286*	Sensor histidine kinase	−3.44	1,224.43
***orf8446***	[Protein-PII] uridylyltransferase	−4.23	1,014.49
*orf8447*	Nitrogen regulatory protein P-II	−4.47	1,014.49
*orf8448*	Ammonium transporter	−4.69	1,014.49
***orf3840***	FIG00815578: hypothetical protein	−2.04	635.43
*orf3841*	Methionine ABC transporter ATP-binding protein	4.49	635.43
*orf3842*	Methionine ABC transporter permease protein	4.79	635.43
*orf3843*	Methionine ABC transporter substrate-binding protein	5.69	635.43
***orf8469***	Chitinase	−4.54	613.74
***orf9470***	Putative glycosyl hydrolase	2.35	366.23
***orf9219***	Phosphate ABC transporter, periplasmic phosphate-binding protein PstS	−7.16	203.59
***orf3212***	Exodeoxyribonuclease III	2.58	111.54
***orf1935***	Probable protein p60 precursor	3.88	103.36

^*a*^ Bold indicates that the most significant promoter regions are in front of the 10 genes.

Subsequently, the MtrA motif was compared with motifs in the prokaryote DNA database using the motif database scanning algorithm TOMTOM (19). As listed in Table 2, it suggested that the MtrA motif (*A. deccanensis*) shows great similarity to the GlnR (*Saccharopolyspora erythraea*) motif, BldD (*S. coelicolor*) motif, and OmpR (*Salmonella enterica*) motif, which implied that these regulator families could participate in competitive or cooperative motif binding with MtrA and co-regulate nitrogen metabolism and the growth of mycelia. To test whether this MtrA motif is suitable for searching binding areas in other promoter regions or species, we uploaded eight proven promoter-binding sequences (Table S7, from *A. deccanensis*, *S. coelicolor,* and *S. venezuelae*) together with the MtrA motif to the FIMO server (13). Matched sequences are shown in Fig. 5D, and predicted sites (covered with translucent yellow) are basically consistent with the former experimental results (*S. coelicolor* and *S. venezuelae*, marked in red). As for the predicted sites in *A. deccanensis*, we deleted 5 or 10 bp of the binding probes, and the EMSA results showed that these probes weakened or lost their ability to be bound by MtrA (Fig. 5E). In general, it suggested that the MtrA motif discovered here is beneficial for finding potential binding sites in actinomycetes.

**TABLE 2:** MtrA motif compared with motifs in the prokaryote DNA database

Motif name	*P*-value	*E*- value	*q*-value	Overlap	Logo
MtrA_top10motif (MtrA_*A.deccanensis*)	0	0	0	20	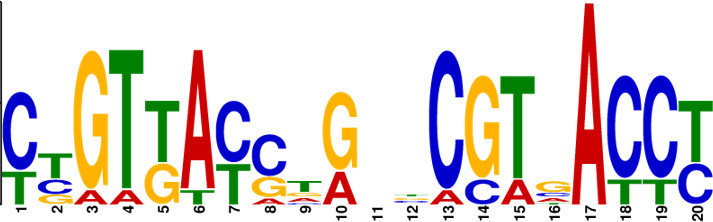
EXPREG_00000200 (GlnR_*S.erythraea*)	6.23e-04	5.23e-02	1.03e-01	16	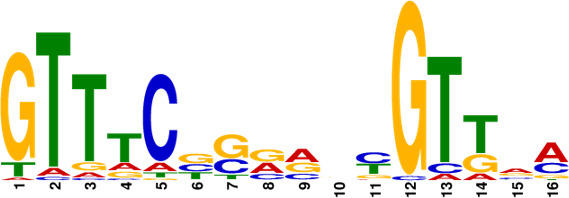
EXPREG_00000fc0 (BldD_*S.coelicolor*)	2.33e-03	1.96e-01	1.94e-01	15	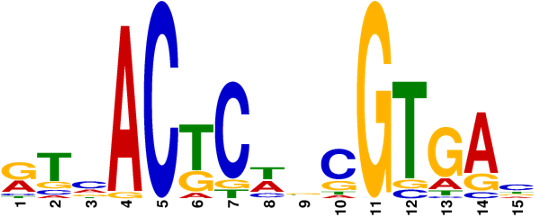
EXPREG_00000810 (OmpR_*S.enterica*)	3.59e-03	3.01e-01	1.98e-01	9	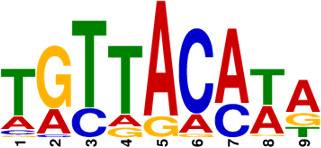
EXPREG_000007b0 (MatP_*E.coli*)	1.25e-02	1.05e + 00	4.62e-01	20	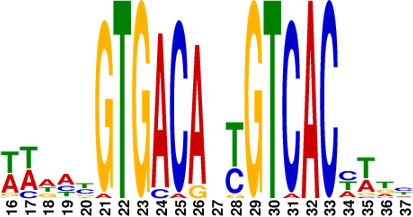
EXPREG_00000870 (RutR_*E.coli*)	1.39e-02	1.17e + 00	4.62e-01	16	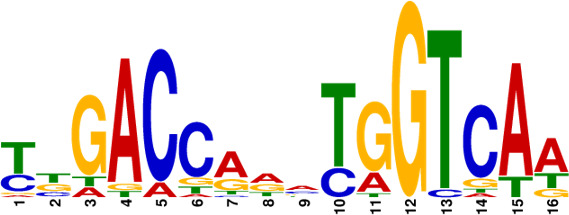

## DISCUSSION

In our study, we used biotinylated *fadS5R1p* to directionally isolate and characterize MtrA in *A. deccanensis* YP-1 as a positive regulator of fidaxomicin production by directly binding to the promoter region of the FDX pathway-specific regulator FadR1. FDX production was improved by 37% through the *mtrA* overexpression strategy and decreased by 61% in the *mtrA* mutant strain. Overall, the location and elucidation of the regulatory role of MtrA enriched the understanding of the entire fidaxomicin biosynthetic network and the investigation of important functions of analogical OmpR family regulators in the gene clusters of other macrolide antibiotics.

MtrA is a response regulator of the two-component signal transduction system MtrAB-lpqB, which is in charge of sensing and responding to environmental stress (Fig. S5). The two-component signal transduction system (TCS) generally consists of a transmembrane histidine kinase (HK) and a cytosolic response regulator (RR) (20). Generally, HK senses an external stimulus that results in ATP hydrolysis and phosphorylation of the conservative histidine residues of HK. Subsequently, phosphorylated HK transfers the phosphoryl group to the conservative aspartic acid residue of homologous RR, which ultimately leads to the activation of a downstream effector domain that elicits the specific response (21, 22). Most of the known TCS involve the regulation of bacterial growth and development and secondary metabolism (23 – 25). The response regulator MtrA (*Mycobacterium tuberculosis* regulator A) was first characterized as an essential gene regulating cell replication, cell wall synthesis, and adaptation to environmental changes in the actinomycete MTB, and knockout strains of *mtrA* are unavailable (26 – 28). In *Streptomyces coelicolor*, MtrA binds to the promoter regions of developmental genes and the genes encoding ActII-1, ActII-4, and RedZ, which are cluster-situated regulators of the antibiotics actinorhodin and undecylprodigiosin (29). In *Streptomyces venezuelae*, MtrA positively regulates jadomycin production but represses chloramphenicol production (13). Besides the impact on secondary metabolites, the MtrAB TCS also directly or indirectly affects osmoprotection, cell wall homeostasis, cell cycle progression, and the developmental life cycle (30 – 32).

Notably, deletion of MtrA not only greatly deteriorated the production of FDX but also affected the growth of mycelia, resistance to cell envelope stress, pigment biosynthesis, efficiency of conjugation, etc. For a comprehensive understanding of MtrA as an important pleiotropic regulator, combined with DNA affinity purification and high-throughput sequencing, we revealed that MtrA directly binds to the genes encoding oligopeptide-binding protein OppA (*orf9414*), PQQ-binding-like beta-propeller repeat protein (*orf8222*), transglycosylase, nitrogen regulatory protein P-II (*orf8447*), ammonium transporter (*orf8448*), methionine ABC transporters (*orf3841*, *orf3842*, and *orf3843*), chitinase (*orf8469*), glycosyl hydrolase (*orf9470*), phosphate transporter (*orf9219*), exodeoxyribonuclease III (*orf3212*), drug transport proteins (*orf459* and *orf5652*), and outer membrane stress sensor (*orf9266*). Thus, MtrA participates in the global regulation of cell division, peptidoglycan synthesis, drug transport, phosphate transport, methionine transport, nitrogen metabolism, and secondary metabolism.

For analysis of the MtrA regulatory mechanism, the MtrA binding sites must be known. Due to the difficulty of mining potential binding probes, the limited number of samples and weak strength of binding usually cause deviation. With the aid of RNA-seq and DAP-seq, we screened out the 10 most significant and strong binding areas among fragments of the whole YP-1 genome and summarized that MtrA tends to bind CYGTKAYSTGNBCGTGACCY containing two short GTKAYS-rich motifs separated by five base pairs, which can be used as a tool to excavate potential MtrA sites in actinomycetes. By comparison in TOMTOM, we found that the binding motifs of MtrA, GlnR (nitrogen metabolism regulator), and BldD (morphological development regulator) share great similarities with each other. This indicated that there can be some regulatory interaction mechanism between them.

In general, locating and analyzing the pathway-specific regulators in certain biosynthetic gene clusters are relatively simple, but the environmental signals and signal transduction systems that control the expression of gene clusters are poorly understood, which is why most of the gene clusters remain silent. Deletion of MtrA directly hindered FDX biosynthesis and indirectly activated orange pigment production, which showed the great potential of MtrA as an important pleiotropic regulator to activate cryptic gene clusters in actinomycetes. For instance, according to the conserved binding sites CYGTKAYSTGNBCGTGACCY of MtrA, we can perform whole genome scanning to locate the conserved sites by the MEME Suite-FIMO tool and predict the target cryptic gene clusters. Then, the MtrA regulator can be expressed or deleted in the corresponding host to activate silent gene clusters. Similar to other actinomycetes, deletion of MtrA caused changes in antibiotic production, hyperosmotic tolerance, alkali resistance, phosphate transport, and nitrogen metabolism. Here, in the FDX industrial production bacterium *Actinoplanes deccanensis* YP-1, we directionally screened out MtrA as an important global regulator of FDX biosynthesis and explored its role in morphology changes, CDW changes, and efficiency of conjugation. Meanwhile, we tested and verified the conserved binding sites with higher confidence.

In summary, this study not only puts up an effective strategy to improve the yield of FDX but also sets the stage for an increased understanding of the MtrA regulatory network, which would have general implications for OmpR family regulators in actinobacteria. Based on actinomycetes, our study applied a bottom-up (DNA to proteins) regulator excavating strategy and provided a new angle for directionally activating antibiotic production. With top-town (protein to DNAs) regulator characterization methods, we proposed a new feasible scheme for excavating candidate genes and conserved motifs.

## MATERIALS AND METHODS

### Strains, plasmids, primer pairs, and growth conditions

All bacterial strains and plasmids used in this study are listed in Table S1. Growth conditions for *A. deccanensis* and *Escherichia coli* strains were described previously (33). ISP4 was used for *A. deccanensis* growth and phenotype observation. YEME was used as the seed medium and fermentation medium for FDX production.

### Affinity isolation of *fadS5R1p*-interactive proteins and MtrA-interactive motifs

The strategy of finding *fadS5R1p*-interactive proteins was performed by DNA affinity experiments according to a previously described method (34). Here, the *fadS5R1p* fragment amplified using fadS5R1p-F and fadS5R1p-R was cloned into pTA2. The vector probe and V-*fadS5R1p* probe were prepared by PCR with primers pTA2-F-biotin and PTA2-R. Finally, the target protein-digested samples were loaded into LC/MS/MS, and later experiments and data analysis were completed by the staff in the Analysis Center of Agrobiology and Environmental Sciences.

DNA affinity purification sequencing (DAP-seq) experiments and data analysis were performed by the staff in Zoonbio Biotechnology (Nanjing, China). The halo-tagged MtrA regulator was expressed using the TNT (transcription/translation) system (Promega), and the purified protein was then incubated with the adaptor-ligated genomic DNA library. Purified DNA libraries are sequenced using next-generation sequencing, and the resulting genome-wide binding events are analyzed by the MEME Suite (https://meme-suite.org/meme/) and visualized by Tablet and IGV_2.16.0 software (35 – 38).

### Electrophoretic mobility shift assays

The primer pair 28a-*EcoRI-mtrA*-F and 28a-*HindIII-mtrA*-R (Table S2) was used to amplify the *mtrA* gene. The *mtrA* fragment was cloned into pET28a (+) to get the expression plasmid pET28a-*mtrA*, which was then introduced into *E. coli* BL21 (DE3). The resulting strain was grown at 37°C to an optical density (OD_600_) of 0.5–0.8 and then induced with 0.2 mM isopropyl-beta-D-thiogalactopyranoside (IPTG) at 16°C and 160 rpm for 20 h. The soluble histidine-tagged MtrA was purified with Ni^2+^-nitrilotriacetic acid (NTA) resin (Qiagen) according to the manufacturer’s instructions.

Probes carrying promoter regions of tested genes were amplified using the corresponding primer pair (Table S2), with pTA2-F-fam and pTA2-R, and EMSAs were performed as described previously (16).

### DNase I footprinting assay

To identify binding sites of MtrA, 5′-FAM fluorescence-labeled DNA fragments corresponding to upstream regions of tested genes were PCR-synthesized using primers listed in Table S2 and gel-purified. The DNase I footprinting assays were carried out as previously described (16), the DNA samples were loaded into an ABI 3730XL sequencer, and the electropherogram data were analyzed using the software program GeneMarker V2.6.3.

### Dry weight determination

The strain was cultured in YEME seed medium for 2 days and transferred to YEME fermentation medium, making sure the final OD_600nm_ = 0.35. Mycelia were collected from day 1 to day 10, respectively, and placed in a metal bath at 65°C for 3 days until the water was completely evaporated. The dry weight was measured with an electronic balance (dry weight = sample tube − empty tube; *n* = 3).

### Observation of phenotypes

The phenotype of hyphae was observed by SEM. The samples were fixed with 2.5% glutaraldehyde in 0.1 M phosphate buffer (pH = 7.4) (PBS) overnight. Then, samples were washed three times with PBS (soaked for 10 min) and then fixed with 1% OsO_4_ for 2 h. Next, OsO_4_ was removed by washing three times with PBS. In order to dehydrate samples, different gradients of ethanol solution (30%, 50%, 70%, 80%, 90%, 95%, and 100%) were added and soaked for 10 min each time. Finally, the samples were dried through the critical point, and the coating and SEM observation procedures were completed by the staff in the ZJU Center of Cryo-Electron Microscopy (ZJU-CCEM).

### In-frame deletion and complementation of MtrA

The CRISPR/Cpf1-mediated genome editing method was used to construct Δ*mtrA* and other deletion mutants. First, the *A. deccanensis* YP-1 genome data were uploaded to CHOPCHOP (https://chopchop.cbu.uib.no/), and 23-nt protospacer sequences were designed by choosing the knockout region (39). The protospacer with the highest rank on the website was selected to construct the editing plasmid. Using pKCCpf1 as the template, the sgRNA expression cassettes were obtained by PCR with the forward primer 5′-N23-atctacaacagtagaaatttggccacg-3′ (N23 represents the 23-nt protospacer sequences) and the reverse primer (sgRNA-R). Two homologous arms were also amplified with overlaps, and then two homologous arms, sgRNA and *NdeI*/*SpeI*-digested pKCCpf1, are ligated together by the ClonExpress MultiS One Step Cloning Kit (Vazyme Biotech Co., China). The successfully built plasmids were introduced into ET12567/pUZ8002. After conjugal transfer with *A. deccanensis*, exconjugants were selected on MS-ISP4 plates with apramycin and nalidixic acid. To remove the plasmid with a resistance tag, knockout strains were grown on ISP4 medium without apramycin at 37°C for two rounds.

### RT-qPCR and comparative transcriptome analysis

*A. deccanensis* YP-1 wild-type and *A. deccanensis* Δ*mtrA* mutant strains were grown in YEME at 220 rpm and 30°C. The cells from three biological replicates were collected at 48 and 96 h and treated with RNA protection, and afterward, RNA extraction, library construction, transcriptome identification, and analysis of WT, Δ*mtrA,* and Δ*mtrAB-lpqB* strains were demonstrated by GENEWIZ (Suzhou, China).

### HPLC assay of fidaxomicin

The fermentation broth was treated with four volumes of methanol and centrifuged. The supernatants were collected and filtered through a Millipore membrane for HPLC analysis. The secondary metabolites were analyzed using the LC-20AT System (Shimadzu) with the method described before (33). Pure fidaxomicin was used as a standard. The UV detection wavelength was set at 254 nm.

## Data Availability

The transcriptome data have been deposited in the Sequence Read Archive (SRA) (https://www.ncbi.nlm.nih.gov/sra) under accession number PRJNA903288.

## SUPPLEMENTAL MATERIAL

Supplemental material spectrum.02702-23-s0001.docx Fig. S1 to S5; Tables S1 to S7.

ASM does not own the copyrights to Supplemental Material that may be linked to, or accessed through, an article. The authors have granted ASM a non-exclusive, world-wide license to publish the Supplemental Material files. Please contact the corresponding author directly for reuse.
