# Supplementary material for: Transcriptional regulation of the fidaxomicin gene cluster and cellular development in Actinoplanes deccanensis YP-1 by the pleiotropic regulator MtrA
Source: Microbiol Spectr. 2023 Nov 15;11(6):e02702-23. doi: 10.1128/spectrum.02702-23 (PMC10714768; doi:10.1128/spectrum.02702-23)
Supplement: Supplemental material — Fig. S1 to S5; Tables S1 to S7. [file spectrum.02702-23-s0001.docx]

**Transcriptional regulation of the fidaxomicin gene cluster and cellular development in** ***Actinoplanes deccanensis* YP-1 by a pleiotropic regulator, MtrA**

Huang Xie^a,b^, Jing-Yi Ruan^a,b^, Qing-Ting Bu^a,b^, Yue-Ping Li^a,b^, Yi-Ting Su^a,b^, Qing-Wei Zhao^a^ , Yi-Ling Du^a,b^#, Yong-Quan Li ^a,b^ #

-The affilication(s) and address(es) of the author(s): ^a^ First Affiliated Hospital and

Institute of Pharmaceutical Biotechnology, Zhejiang University School of Medicine,

Hangzhou310058, China.

^b^ Zhejiang Provincial Key Laboratory for Microbial Biochemistry and Metabolic Engineering, Hangzhou310058, China.

-The e-mail address, telephone and fax numbers of the corresponding author:

*Correspondence: Yong-Quan Li, E-mail: lyq@zju.edu.cn. Tel.: +86-0571-88206632

Fax: +86 0571-88208569

**Supplementary Results**


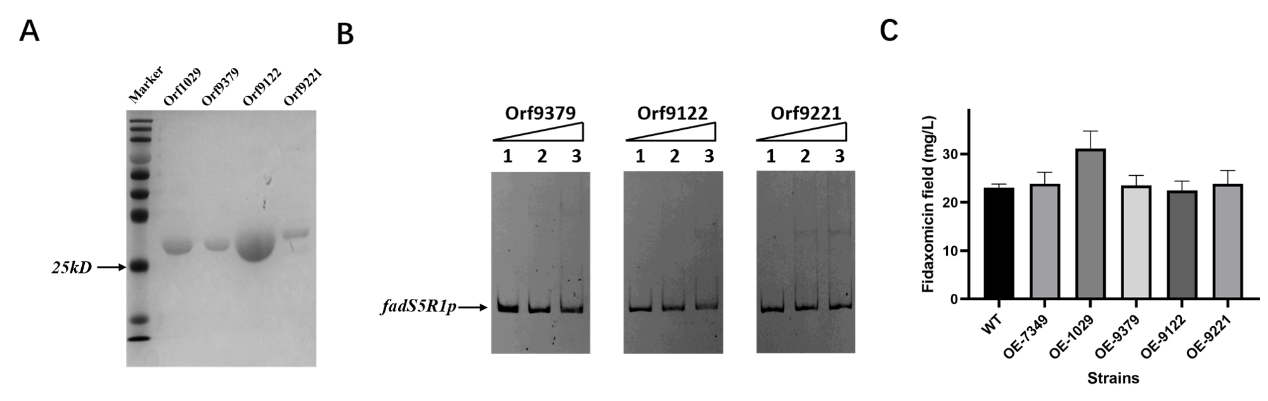


**Fig. S1** Identification of functional proteins and MtrA-*fadS5R1p* binding sites.

(A) Purified regulatory proteins. M replaces to 10-170 KDa Prestained Protein Ladder (Thermo).

(B) EMSA with screened regulators and *fadS5R1p*. Reactions were carried out with the addition of no MtrA (lane 1), or with 0.025μg (lane 2), 0.05μg (lane 3) MtrA in 10μL mix.

(C) FDX field in different regulator-overexpressed strains.


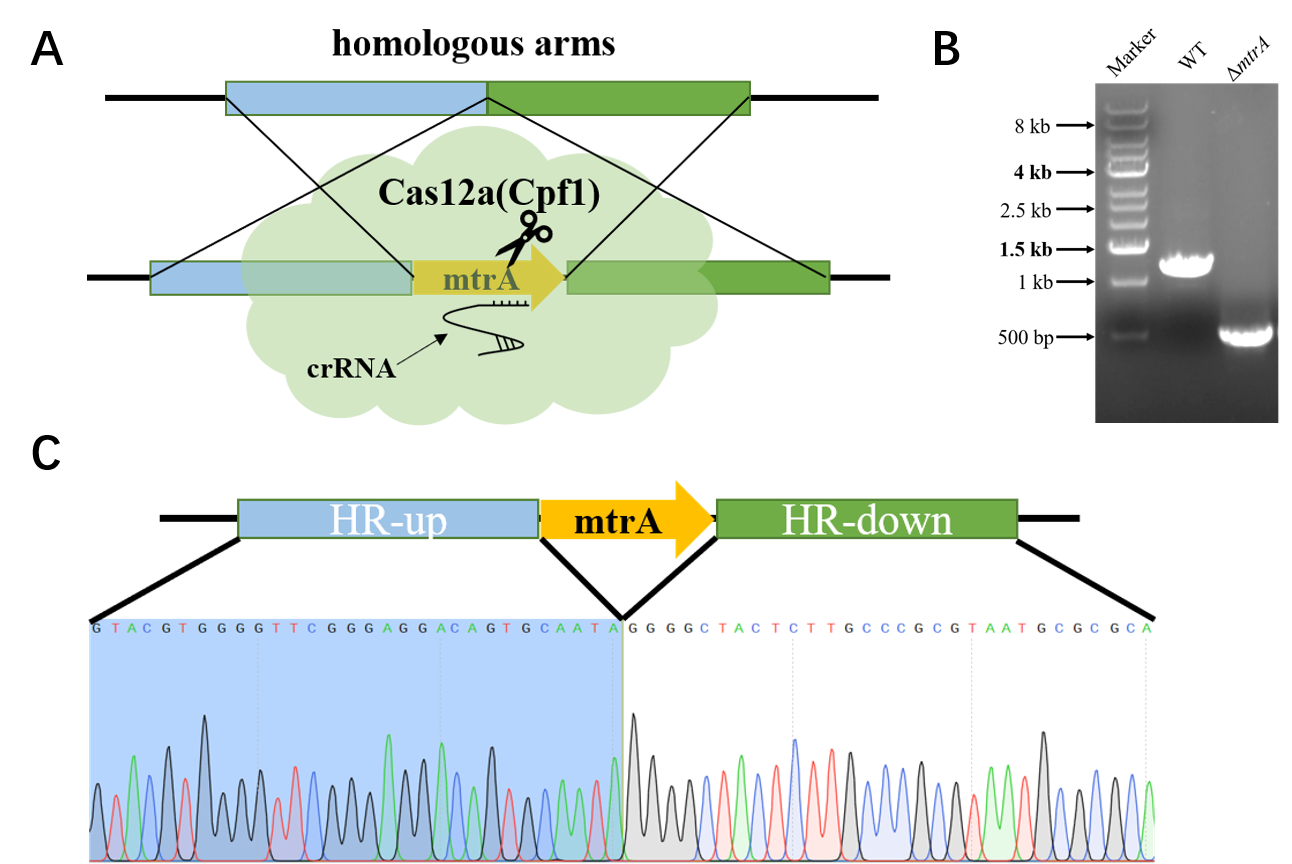


**Fig. S2** Deletion of mtrA and identification of the mutant strain.

(A) Site-specific cleavage by Cas12a/crRNA.

(B) Identification of the mtrA deletion by PCR.

(C) Analysis of mtrA deletion by sequencing.


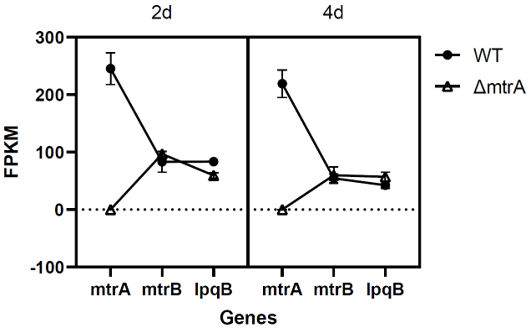


**Fig. S3** FPKMs of mtrA, mtrB, lpqB in wild-type strain and Δ*mtrA* strain.


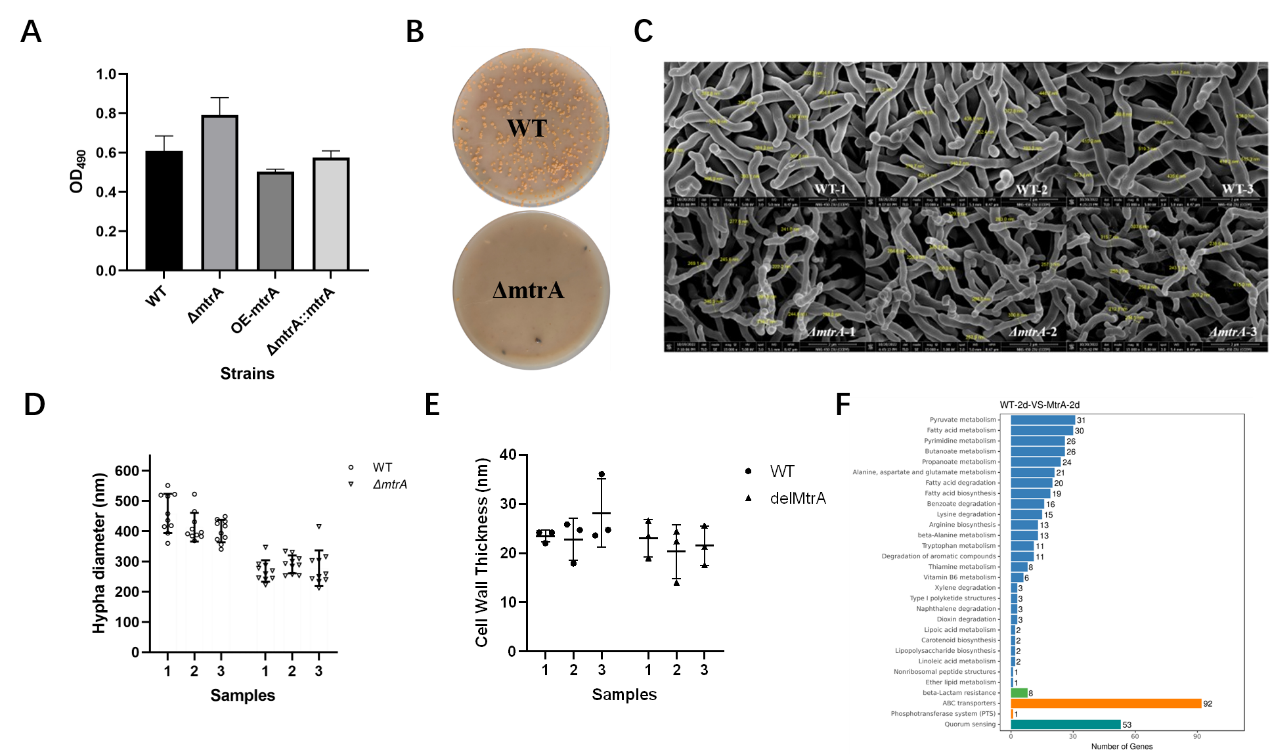


**Fig. S4** Influence of mtrA deletion on orange piment production, conjugation, mycelium development and KEGG pathway.

(A) OD_490_ from WT, Δ*mtrA*, OE-mtrA and Δ*mtrA*::*mtrA* fermentation experiments (n = 3, mean with SD).

(B) Conjugation ISP4-MS (20mM Mg^2+^) plates of WT and Δ*mtrA* (provided with the same concentration of actinomyces mycelium and ET12567/pUZ8002/pIJ8660-mcs).

(C) Scanning electron micrographs of *Actinoplanes deccanensis* YP-1 wild type and the Δ*mtrA* mutant after cultivation for 2d on ISP4 agar plates. The white bar represents a length of 2 μm.

(D) Analysis of hypha diameter based on SEM results.

(E) Analysis of cell envelope thickness based on TEM results. Image-Pro software was used for data statistics.

(F) Distribution statistics of differential gene enrichment in KEGG pathways in WT and Δ*mtrA* transcriptomes (48 h).


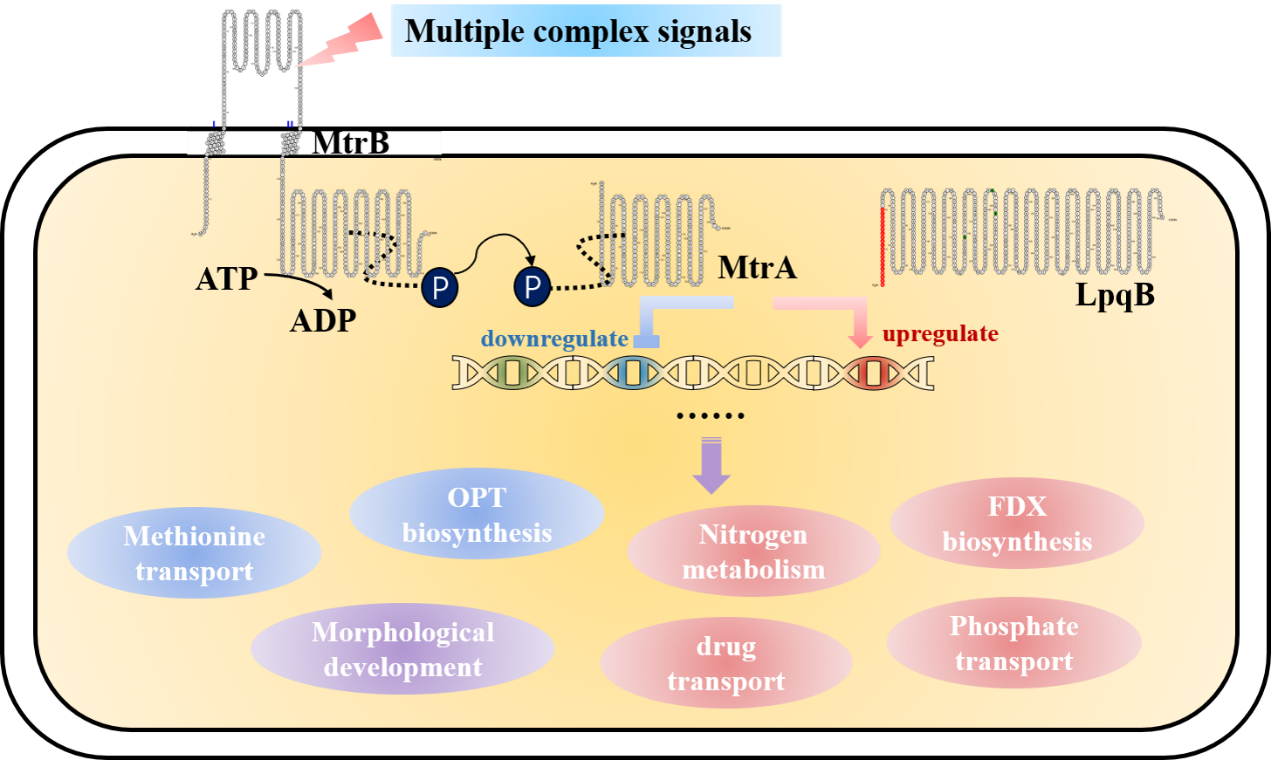


**Fig. S5** Action mode of MtrAB-LpqB dealing with environmental stress.

**Table S1. Strains and plasmids used in this study**

| Strain or plasmid | | Genotype and description | | Source and reference | |
| --- | --- | --- | --- | --- | --- |
| ***strains*** | |  | |  | |
| *Actinoplanes deccanensis* YP-1 | | Wild-type producer of fidaxomicin | | CGMCC 4.2098 | |
| YP-1 Δ*mtrA* | | YP-1 *mtrA* deletion mutant | | This study | |
| YP-1 OE-*mtrA* | | YP-1 harboring pSOK804-*ermEp**-*mtrA*; Apra^r^ | | This study | |
| YP-1 OE-*mtrB* | | YP-1 harboring pSOK804-*ermEp**-*mtrB*; Apra^r^ | | This study | |
| YP-1 OE-*mtrAB* | | YP-1 harboring pSOK804-*ermEp**-*mtrAB*; Apra^r^ | | This study | |
| YP-1 OE-*lpqB* | | YP-1 harboring pSOK804-*ermEp**-*lpqB*; Apra^r^ | | This study | |
| YP-1 OE-*bldD* | | YP-1 harboring pSOK804-*ermEp**-*bldD*; Apra^r^ | | This study | |
| YP-1 OE-*orf9379* | | YP-1 harboring pSOK804-*ermEp**-*orf9379*; Apra^r^ | | This study | |
| YP-1 OE-*phoU* | | YP-1 harboring pSOK804-*ermEp**-*phoU*; Apra^r^ | | This study | |
| YP-1 OE-*glnR* | | YP-1 harboring pSOK804-*ermEp**-*glnR*; Apra^r^ | | This study | |
| YP-1 Δ*mtrA*::*mtrA* | | *ΔmtrA* harboring pSOK804-*mtrAp*-*mtrA*; Apra^r^ | | This study | |
| YP-1 Δ*mtrAB-lpqB* | | YP-1 *mtrA*, *mtrB* and *lpqB* deletion mutant | | This study | |
| YP-1 Δ*mtrB* | | YP-1 *mtrB* deletion mutant | | This study | |
| ***Escherichia coli*** | |  | |  | |
| DH5α | | General cloning host | | Tsingke | |
| BL21(DE3) | | Strain for protein expression | | Invitrogen | |
| ET12567/pUZ8002 | | ET12567 containing the non-transmissible RP4 derivative plasmid pUZ8002; helper strain for intergeneric conjugation; | | Invitrogen | |
| ***plasmids*** | |  | |  | |
| pTA2 | | General cloning vector | | TOYOBO | |
| pET28a (+) | | Protein expression vector | | Invitrogen | |
| pET28a-*bldD* | | Construct for expression of BldD | | This study | |
| pET28a-*mtrA* | | Construct for expression of MtrA | | This study | |
| pET28a-*orf9379* | | Construct for expression of Orf9379 | | This study | |
| pET28a-*phoU* | | Construct for expression of PhoU | | This study | |
| pET28a-*glnR* | | Construct for expression of GlnR | | This study | |
| pKCCpf1 | | A vector derived from pKC1139 containing the scocpf1 gene under the control of *ermEp** and the crRNA repeat unit under the control of *kasOp** | | Professor Yinhua Lu’s lab | |
| pKCCpf1-*mtrA* | | pKCCpf1 with the crRNA transcription cassette for deleting *orf1029* (*mtrA*) and two homologous arms | | This study | |
| pKCCpf1-*mtrAB-lpqB* | | pKCCpf1 with the crRNA transcription cassette for deleting *orf1029-1031* (*mtrAB-lpqB*) and two homologous arms | | This study | |
| pKCCpf1-*mtrB* | | pKCCpf1 with the crRNA transcription cassette for deleting *orf1030* (*mtrB*) and two homologous arms | | This study | |
| pSOK804 | | A integrative vector containing *oriT*, VWB *int/attP*; Apra^r^ | | This lab | |
| pSOK804-*ermEp** | | A integrative vector containing *oriT*, VWB *int/attP* and *ermEp**; Apra^r^ | | This lab | |
| pSOK804-*ermEp**-*mtrA* | | pSOK804 with *mtrA* expression under the control of *ermEp** | | This study | |
| pSOK804-*mtrAp*-*mtrA* | | pSOK804 with *mtrA* expression under the control of *mtrA*’s original promoter | | This study | |
| pSOK804-*ermEp**-*mtrB* | | pSOK804 with *mtrB* expression under the control of *ermEp** | | This study | |
| pSOK804-*ermEp**-*mtrAB* | | pSOK804 with *mtrAB* expression under the control of *ermEp** | | This study | |
| pSOK804-*ermEp**-*lpqB* | | pSOK804 with *lpqB* expression under the control of *ermEp** | | This study | |
| pTA2-*fadS5R1p* | | pTA2 with the promoter of *fadR1* | | This study | |
| pTA2-*orf459p* | | pTA2 with the promoter of *orf459* | | This study | |
| pTA2-*orf2614p* | | pTA2 with the promoter of *orf2614* | | This study | |
| pTA2-*orf5652p* | | pTA2 with the promoter of *orf5652* | | This study | |
| pTA2-*orf9266p* | | pTA2 with the promoter of *orf9266* | | This study | |

**Table S2. Primers used in this study**

| Primer | Sequence(5’→3’) | Application |
| --- | --- | --- |
| delmtrA-homoA-F | TACGAGATATCGACGCACTAGTggcctctgacctgcgtgcaag | *mtrA* deletion strain |
| delmtrA-homoA-R | GCAAGAGTAGCCCCtattgcactgtcctcccgaacc |  |
| delmtrA-homoB-F | GAGGACAGTGCAATAggggctactcttgcccgcg |  |
| delmtrA-homoB-R | CCGGGCGGCCCGATGGTGAGCATgcgctgatcgacaggccgag |  |
| mtrA-sgRNA-F | ATGCTCACCATCGGGCCGCCCGGatctacaacagtagaaatttggccacg |  |
| sgRNA-R(NdeI) | acgaactcctggtagatggacatatg |  |
| delMtrA-F250 | gaggtcgccaacgttccgtc | delmtrA verification primers |
| delMtrA-R250 | cgtgagcaggatcgtggcac |  |
| delmtrABQ-homoA-F | TACGAGATATCGACGCACTAGTggcctctgacctgcgtgcaag | *mtrA*, *mtrB* and *lpqB* deletion strain |
| delmtrABQ-homoA-R | GCCAGCAGGCTCAAtattgcactgtcctcccgaacc |  |
| delmtrABQ-homoB-F | GAGGACAGTGCAATAttgagcctgctggcggacct |  |
| delmtrABQ-homoB-R | CGCCTGACCCTGCCGGTGCGCTCgcacgatgcgccagggctg |  |
| mtrABQ-sgRNA-F | GAGCGCACCGGCAGGGTCAGGCGatctacaacagtagaaatttggccacg |  |
| sgRNA-R(NdeI) | acgaactcctggtagatggacatatg |  |
| delmtrB-homoA-F | CCTACGAGATATCGACGCACTAGTgagccgaacggccatcgctg | *mtrB* deletion strain |
| delmtrB-homoA-R | GCAGCCAGCGACGCATacgccaataccgccgcacc |  |
| delmtrB-homoB-F | CGGCGGTATTGGCGTtgcgtcgctggctgctgg |  |
| delmtrB-homoB-R | GGCTGGTTCATCGCCGCGCGCAGcaccgagatcgcggtgatcg |  |
| mtrB-sgRNA-F | CTGCGCGCGGCGATGAACCAGCCatctacaacagtagaaatttggccacg |  |
| sgRNA-R(NdeI) | acgaactcctggtagatggacatatg |  |
| 804-ep-mtrA-F | GGAGGCGGACATCATATGttggaccgcatgagagcccg | *mtrA* overexpressed strain |
| 804-ep-mtrA-R | TGACATGATTACGAATTCttagcccgtgcccgccttg |  |
| 804-ori-mtrA-F | CGCCTCATGGAGGGCGCGAAGCTTcctcaggtccggggcggc | *mtrA* complementary strain |
| 804-ori-mtrA-R | GACATGATTACGAATTCGATATCttagcccgtgcccgccttgtag |  |
| 804-ep-mtrB-F | GGAGGCGGACATCATATGgtggccgcgcggcggctcg | *mtrB* overexpressed strain |
| 804-ep-mtrB-R | TGACATGATTACGAATTCtcaggacacctccggggcctc |  |
| 804-ep-mtrAB-F | GGAGGCGGACATCATATGttggaccgcatgagagcccg | *mtrAB* overexpressed strain |
| 804-ep-mtrAB-R | TGACATGATTACGAATTCtcaggacacctccggggcct |  |
| 804-ep-lpqB-F | GGAGGCGGACATCATATGatgcgtcgctggctgctggg | *lpqB* overexpressed strain |
| 804-ep-lpqB-R | TGACATGATTACGAATTCtcagttgaggaacagcggcgtg |  |
| 28a-EcoRI-bldD-F | TGGGTCGCGGATCCGAATTCatgccgtctgagtacgccaa | BldD protein expression |
| 28a-HindIII-bldD-R | TCGAGTGCGGCCGCAAGCTTtcagctggcgaagaacgcc |  |
| 28a-EcoRI-mtrA-F | TGGGTCGCGGATCCGAATTCttggaccgcatgagagcccg | MtrA protein expression |
| 28a-HindIII-mtrA-R | CGAGTGCGGCCGCAAGCTTttagcccgtgcccgccttg |  |
| 28a-EcoRI-orf9379-F | TGGGTCGCGGATCCGAATTCatggatgaggtactggcgcg | Orf9379 protein expression |
| 28a-HindIII-orf9379-R | CGAGTGCGGCCGCAAGCTTtcagacacgcgcgcgccg |  |
| 28a-EcoRI-phoU-F | TGGGTCGCGGATCCGAATTCatgcgcgaggagttccagg | PhoU protein expression |
| 28a-HindIII-phoU-R | CGAGTGCGGCCGCAAGCTTtcagctctggtcggtcacctg |  |
| 28a-EcoRI-glnR-F | TGGGTCGCGGATCCGAATTCgtggaaatccttctgttggtgacgg | GlnR protein expression |
| 28a-HindIII-glnR-R | CGAGTGCGGCCGCAAGCTTtcagacgggcagcgagac |  |
| pTA2-F-fam | gcttgatatcgaattcccaat (5’-FAM) | EMSA |
| pTA2-F-botin | gcttgatatcgaattcccaat (5’-BOTIN) | DNA-protein affinity |
| pTA2-R | gggctgcaggaattcccaat |  |
| fadS5R1p-F | ggtgcctccgtggtcgtc | probe amplification |
| fadS5R1p-R | tctgctatccatcaagccaac |  |
| fadS5R1p-R1-mut | aatccacgcagtggcaacgaatcgctcaactccccg | probe mutation |
| fadS5R1p-F2-mut | cgttgccactgcgtggattctgggaatccgtggcccctac |  |
| fadS5R1p-R1-del16 | gtaggggccacggattcccagacgaatcgctcaactccccg | probe truncation |
| fadS5R1p-F2-del16 | cggggagttgagcgattcgtctgggaatccgtggcccctac |  |
| orf459p-F | cctgcatggtgacattccgg | probes amplification |
| orf459p-R | cagcgcgacccacttgtaac |  |
| orf2614p-F | gcctgcatcgtcgcacgtg |  |
| orf2614p-R | ggacaagaccgcctcctcga |  |
| orf5652p-F | gccttgggctgcttgaacg |  |
| orf5652p-R | gtgatcggcggtccgcgc |  |
| orf9266p-F | ccggaatcttccgatcgatctg |  |
| orf9266p-R | tatcgataaggcgcgcgcga |  |
| qRT-hrdB-F | gacgacgaggagtccgagg | FDX-BGC qRT-PCR primers |
| qRT-hrdB-R | ttgagcagcgggaccttg |  |
| qRT-I-F | cgctgttgtcaagactttcctc |  |
| qRT-I-R | cgtgctgtggagctgggtc |  |
| qRT-T1-F | ggcgtacacctggatcgtca |  |
| qRT-T1-R | cctgcaacagccacttcttgtc |  |
| qRT-S4-F | cggagatgttcggcaaggt |  |
| qRT-S4-R | gtgatgaagtgcccgtaggc |  |
| qRT-G2-F | tgatcgtggccgactcgtg |  |
| qRT-G2-R | accccggtgtcggatagct |  |
| qRT-B-F | ggtgtcttcgtcgggctgtg |  |
| qRT-B-R | cgtgcgagatccggttgg |  |
| qRT-F-F | aggcggtcaagcagttcgg |  |
| qRT-F-R | ggtcagatggcgttgcaggt |  |
| qRT-E-F | gctgccggtgcagtatcc |  |
| qRT-E-R | tgctgtgcccgaagaacg |  |
| qRT-D-F | caccacagcgagtcgaacg |  |
| qRT-D-R | gcggcacttgggctcataga |  |
| qRT-C-F | ccacgtgttccccagctatc |  |
| qRT-C-R | ggctgtagatgtggaagcgg |  |
| qRT-S2-F | gcggcatgaccatgaactg |  |
| qRT-S2-R | cctcgtccggtgtcacaaa |  |
| qRT-S1-F | ctccaagggttacgaggtctg |  |
| qRT-S1-R | ccgtattcgaggtgcagtcc |  |
| qRT-G1-F | aacctgctgcccacgctg |  |
| qRT-G1-R | gcccgaagacggagaagacc |  |
| qRT-T2-F | agttcaaccccgcccagc |  |
| qRT-T2-R | cgaactccgtgaagccgac |  |
| qRT-W3-F | ggcaagtccaccctcatcga |  |
| qRT-W3-R | gtcgccgaggatgctgcc |  |
| qRT-W2-F | aggagtcgaacgccttctac |  |
| qRT-W2-R | ccgcatgtcgagcagggag |  |
| qRT-W1-F | agcacgacggctcggacc |  |
| qRT-W1-R | cccggtcgtacggaaaggtc |  |
| qRT-Y2-F | ggacttcaaatccgatcagg |  |
| qRT-Y2-R | tacgggaaccgtgaggagc |  |
| qRT-Y1-F | ggctggattgggtggtcat |  |
| qRT-Y1-R | cacgaatctcggcaggaaaa |  |
| qRT-N-F | cggttgaccgcccactatgt |  |
| qRT-N-R | ccggagaaggtcggccat |  |
| qRT-A4-F | cttcgacgccgcgttcttc |  |
| qRT-A4-R | cggtcgcgtagtcgtgatag |  |
| qRT-A3-F | gaacacgtcgcaggagcaa |  |
| qRT-A3-R | ccagcagcgcccacagat |  |
| qRT-A2-F | acgcggagttcttcgggatc |  |
| qRT-A2-R | cgtgcggcgtagtcgtggta |  |
| qRT-A1-F | cagaacaattcctgccgtg |  |
| qRT-A1-R | agggcgtgcgccacctct |  |
| qRT-P2-F | tccgccggaaccgttgaa |  |
| qRT-P2-R | ggcagcgaaatgggtgagc |  |
| qRT-S6-F | gcctccagcgtcaacatcg |  |
| qRT-S6-R | cgcccagtaccacagtctcc |  |
| qRT-M-F | ccacgtcaagcgggttctg |  |
| qRT-M-R | gtcgccggttgttgtagtgc |  |
| qRT-R1-F | tgctgaggcaccaccactg |  |
| qRT-R1-R | tgagccgatggatctccg |  |
| qRT-S5-F | cgccgatctgacaaacgc |  |
| qRT-S5-R | aacctcctggtacagcaact |  |
| qRT-P1-F | tcatggcgggctacgagac |  |
| qRT-P1-R | cggtggacagcagcgaggt |  |
| qRT-L-F | gtgatcgggctctggcact |  |
| qRT-L-R | gcccaggacgaccgagat |  |
| qRT-K-F | atgaatcgtgctttcgtgga |  |
| qRT-K-R | acatctcctgctcctcctcg |  |
| qRT-J-F | atgtcgtcgaacgcctcca |  |
| qRT-J-R | gcgacggtaacccctacaagc |  |

**Table S3. Annotation of top5 screened regulators in LC-MS/MS**

| Gene | Description | Score | Coverage | # Peptides | # PSMs | MW [kDa] |
| --- | --- | --- | --- | --- | --- | --- |
| *orf7349* | Pleiotropic negative regulator BldD | 107.32 | 54.32 | 9 | 34 | 17.9 |
| *orf1029* | Two component system response regulator MtrA | 55.16 | 40.52 | 7 | 19 | 25.6 |
| *orf9379* | catabolite gene activator and cAMP-binding protein kinase | 48.05 | 43.56 | 7 | 14 | 24.7 |
| *orf9122* | Phosphate transport system regulatory protein PhoU | 42.01 | 27.19 | 4 | 12 | 23.8 |
| *orf9221* | Transcriptional regulatory protein GlnR | 35.46 | 26.05 | 4 | 11 | 25.6 |

**Table S4. Annotation of some mentioned genes**

| Gene ID | Gene name | RAST annotation | Function |
| --- | --- | --- | --- |
| BGCs |  |  |  |
| *orf161* | *optA* | Phytoene synthase (EC 2.5.1.32) | core gene of BGC-1 (OPT) |
| *orf1251* | *lanB* | Lanthionine biosynthesis protein LanB | core gene of BGC-2 (lanthipeptide-class-i) |
| *orf2108* | NA | Uncharacterized protein conserved in bacteria, NMA0228-like | core gene of BGC-3 (RiPP-like) |
| *orf2417* | NA | hypothetical protein | core gene of BGC-4 (melanin) |
| *orf3824* | NA | hypothetical protein | core gene of BGC-5 (indole) |
| *orf4047* | NA | Coenzyme PQQ synthesis protein E | core gene of BGC-6 (ranthipeptide) |
| *orf4225* | NA | Lycopene beta cyclase (EC 1.14.-.-) | core gene of BGC-7 (terpene) |
| *orf4260* | NA | Siderophore biosynthesis non-ribosomal peptide synthetase modules | core gene of BGC-8 (NRPS) |
| *orf4758* | NA | hypothetical protein | core gene of BGC-9 (siderophore) |
| *orf6562* | NA | non-ribosomal peptide synthetase, terminal component | core gene of BGC-10 (NAPAA) |
| *orf6750* | NA | putative non-ribosomal peptide synthetase | core gene of BGC-11 (NRPS) |
| *orf6836* | NA | Malonyl CoA-acyl carrier protein transacylase (EC 2.3.1.39) | core gene of BGC-12-1 (T1PKS) |
| *orf6879* | NA | 3-oxoacyl-[acyl-carrier-protein] synthase, KASIII (EC 2.3.1.41) | core gene of BGC-12-2 (PKS-like) |
| *orf7027* | *fadA1* | Malonyl CoA-acyl carrier protein transacylase (EC 2.3.1.39) | core gene of BGC-13-1 (FDX) |
| *orf7055* | NA | Malonyl CoA-acyl carrier protein transacylase (EC 2.3.1.39) | core gene of BGC-13-2 (deccanlifehrin) |
| *orf9305* | NA | Chalcone synthase (EC 2.3.1.74) | core gene of BGC-14 (T3PKS) |
| ***Aerial mycelium*** |  |  |  |
| *orf7936* | *ramA* | Lipid A export ATP-binding/permease protein MsbA | mycelium development |
| *orf568* | *ramB* | ABC transporter ATP-binding protein | mycelium development |
| *orf6809* | *ramR* | putative two-component system response regulator | mycelium development |
| *orf6161* | *rdlA* | hypothetical protein | mycelium development |
| *orf2414* | *rdlB* | putative large secreted protein | mycelium development |
| ***Cell division*** |  |  |  |
| *orf1948* | *ftsZ* | Cell division protein FtsZ (EC 3.4.24.-) | Cell division protein |
| *orf1947* | *ftsQ* | Cell division protein FtsQ | Cell division protein |
| *orf1944* | *ftsW* | Cell division protein FtsW | Cell division protein |
| *orf1940* | *ftsI* | Cell division protein FtsI [Peptidoglycan synthetase] (EC 2.4.1.129) | Cell division protein |
| *orf1939* | *ftsL* | Cell division protein FtsL | Cell division protein |
| ***Peptidoglycan*** |  |  |  |
| *orf1816* | *murA* | UDP-N-acetylglucosamine 1-carboxyvinyltransferase | Peptidoglycan biosynthesis |
| *orf9184* | *murB* | UDP-N-acetylenolpyruvoylglucosamine reductase | Peptidoglycan biosynthesis |
| *orf1946* | *murC* | UDP-N-acetylmuramate--alanine ligase | Peptidoglycan biosynthesis |
| *orf2183* | *murD* | UDP-N-acetylmuramoylalanine--D-glutamate ligase | Peptidoglycan biosynthesis |
| *orf1941* | *murE* | UDP-N-acetylmuramoylalanyl-D-glutamate--2,6-diaminopimelate ligase | Peptidoglycan biosynthesis |
| *orf1942* | *murF* | UDP-N-acetylmuramoylalanyl-D-glutamyl-2,6-diaminopimelate--D-alanyl-D-alanine ligase | Peptidoglycan biosynthesis |
| *orf1945* | *murG* | UDP-N-acetylglucosamine--N-acetylmuramyl-(pentapeptide) pyrophosphoryl-undecaprenol N-acetylglucosamine transferase | Peptidoglycan biosynthesis |
| *orf9710* | *murJ* | Proposed peptidoglycan lipid II flippase MurJ | Peptidoglycan biosynthesis |
| *orf1943* | *murX* | Phospho-N-acetylmuramoyl-pentapeptide-transferase | Peptidoglycan biosynthesis |
| *orf4752* | *uppP* | Undecaprenyl-diphosphatase | Peptidoglycan biosynthesis |
| *orf1556* | *glyS* | Glycyl-tRNA synthetase | Peptidoglycan biosynthesis |
| *orf1524* | *uppS* | Undecaprenyl diphosphate synthase | Peptidoglycan biosynthesis |
| *orf51* | *pbp2* | Cell division protein FtsI [Peptidoglycan synthetase] | Class B PBP |
| *orf8478* | *ddlA* | D-alanine--D-alanine ligase (EC 6.3.2.4) | Peptidoglycan biosynthesis |
|  |  |  |  |
| ***MtrAB TCS*** |  |  |  |
| *orf1029* | *mtrA* | Two component sensory transduction transcriptional regulatory protein MtrA | RR of MtrAB |
| *orf1030* | *mtrB* | Sensor histidine kinase MtrB (EC 2.7.3.-) | HK of MtrAB |
| *orf1031* | *lpqB* | LpqB | putative lipoprotein |

**Table S5. Annotation of the genes screened by RNA-seq and DAP-seq**

| Gene ID | RAST annotation | 2day-log2FC^a^ | -log_10_  Pvalue^b^ |
| --- | --- | --- | --- |
| *orf36* | Protein yceI precursor | 4.07 | 7.40 |
| *orf459* | drug transport protein, putative | -7.58 | 6.40 |
| *orf542* | putative keto acyl reductase( EC:1.1.1.- ) | -3.56 | 5.10 |
| *orf755* | Glycine oxidase ThiO (EC 1.4.3.19) | -5.98 | 6.76 |
| *orf756* | Thiamin-phosphate pyrophosphorylase (EC 2.5.1.3) | -4.63 | 6.76 |
| *orf766* | Uroporphyrinogen-III synthase (EC 4.2.1.75) / response regulator | -3.16 | 52.38 |
| *orf791* | Cys-tRNA(Pro) deacylase YbaK | -3.08 | 7.22 |
| *orf792* | Xylose ABC transporter, periplasmic xylose-binding protein XylF | -3.19 | 19.95 |
| *orf793* | D-xylose transport ATP-binding protein XylG | -4.35 | 19.95 |
| *orf794* | Xylose ABC transporter, permease protein XylH | -3.85 | 9.91 |
| *orf1020* | Aldehyde dehydrogenase (EC 1.2.1.3) | 4.20 | 18.23 |
| *orf1205* | Dolichol-phosphate mannosyltransferase (EC 2.4.1.83) in lipid-linked oligosaccharide synthesis cluster | 3.49 | 10.58 |
| *orf1544* | Substrate-specific component YkoE of thiamin-regulated ECF transporter for HydroxyMethylPyrimidine | -5.54 | 6.93 |
| *orf1656* | Maltose/maltodextrin ABC transporter, substrate binding periplasmic protein MalE | 4.80 | 5.08 |
| *orf1771* | Alkaline phosphatase (EC 3.1.3.1) | -3.74 | 6.21 |
| *orf1935* | probable protein p60 precursor | 3.88 | 103.36 |
| *orf2189* | putative sugar transporter sugar binding protein | -3.61 | 22.02 |
| *orf2396* | Transcriptional regulator, IclR family | -4.90 | 68.09 |
| *orf2398* | 2-keto-4-pentenoate hydratase (EC 4.2.1.80) | -7.19 | 68.09 |
| *orf2403* | ABC-type nitrate/sulfonate/bicarbonate transport system, permease component | -8.75 | 9.80 |
| *orf2404* | ABC transporter ATP-binding protein with unknown substrate | -8.62 | 9.80 |
| *orf2435* | Dihydroxyacetone ABC transport system, permease protein 2 # predicted | 3.64 | 5.05 |
| *orf2614* | Urea carboxylase-related amino acid permease | -6.34 | 39.91 |
| *orf2615* | Urea carboxylase-related aminomethyltransferase (EC 2.1.2.10) | -5.50 | 39.91 |
| *orf2921* | Ribonucleotide reductase of class Ia (aerobic), beta subunit (EC 1.17.4.1) | 4.60 | 8.70 |
| *orf2922* | Ribonucleotide reductase of class Ia (aerobic), alpha subunit (EC 1.17.4.1) | 3.88 | 6.62 |
| *orf3098* | Branched-chain amino acid ABC transporter, amino acid-binding protein (TC 3.A.1.4.1) | -10.24 | 20.51 |
| *orf3132* | Dihydropyrimidinase (EC 3.5.2.2) | -7.44 | 8.91 |
| *orf3133* | N-carbamoylputrescine amidase (3.5.1.53) / Omega amidase (Nit2 homolog) | -6.78 | 8.91 |
| *orf3135* | Alanine--glyoxylate aminotransferase 2 homolog 3, mitochondrial precursor (EC 2.6.1.44) (Beta-alanine-pyruvate aminotransferase 3) | -3.30 | 8.91 |
| *orf3212* | Exodeoxyribonuclease III (EC 3.1.11.2) | 2.58 | 111.54 |
| *orf3285* | Multimodular transpeptidase-transglycosylase (EC 2.4.1.129) (EC 3.4.-.-) | -5.66 | 1224.43 |
| *orf3286* | sensor histidine kinase | -3.44 | 1224.43 |
| *orf3482* | probable tetronasin-transport integral membrane protein ABC transporter | -4.14 | 8.26 |
| *orf3840* | FIG00815578: hypothetical protein | -2.04 | 635.43 |
| *orf3841* | Methionine ABC transporter ATP-binding protein | 4.49 | 635.43 |
| *orf3842* | Methionine ABC transporter permease protein | 4.79 | 635.43 |
| *orf3843* | Methionine ABC transporter substrate-binding protein | 5.69 | 635.43 |
| *orf4201* | Two-component response regulator | -3.56 | 6.84 |
| *orf4450* | Colanic acid biosynthesis glycosyl transferase WcaL | -3.46 | 6.06 |
| *orf4462* | L-arabinose transport ATP-binding protein AraG (TC 3.A.1.2.2) | -4.51 | 31.10 |
| *orf4809* | Thiamin ABC transporter, transmembrane component | -5.00 | 7.52 |
| *orf4810* | Sulfate and thiosulfate import ATP-binding protein CysA (EC 3.6.3.25) | -3.88 | 7.52 |
| *orf5253* | putative secreted protein | -2.37 | 43.06 |
| *orf5652* | multidrug-efflux transporter protein | -7.53 | 6.88 |
| *orf5823* | dTDP-Rha:A-D-GlcNAc-diphosphoryl polyprenol, A-3-L-rhamnosyl transferase WbbL | 3.47 | 13.13 |
| *orf6362* | putative glucan-binding protein D; BglB-like protein | 4.59 | 12.57 |
| *orf6374* | Aminopeptidase Y (Arg, Lys, Leu preference) (EC 3.4.11.15) | 4.10 | 12.53 |
| *orf6431* | N-methylhydantoinase A (EC 3.5.2.14) | -4.27 | 7.23 |
| *orf6724* | probable regulatory protein | -4.15 | 9.32 |
| *orf6965* | NAD-dependent formate dehydrogenase beta subunit | 4.26 | 17.55 |
| *orf7031* | LuxR-family transcriptional regulator | -3.44 | 8.74 |
| *orf7032* | Methyltransferase | -4.55 | 8.74 |
| *orf7406* | D-alanyl-D-alanine carboxypeptidase (EC 3.4.16.4) | 4.27 | 7.67 |
| *orf7458* | probable 3-chlorobenzoate-3,4-dioxygenase dyhydrogenase related protein-putative NAD-dependent oxidoreductase | 3.88 | 8.16 |
| *orf7976* | Dipeptide transport system permease protein DppC (TC 3.A.1.5.2) | -4.77 | 9.77 |
| *orf7989* | diguanylate cyclase/phosphodiesterase (GGDEF & EAL domains) with PAS/PAC sensor(s) | 4.54 | 8.36 |
| *orf8024* | Pyruvate decarboxylase (EC 4.1.1.1); Alpha-keto-acid decarboxylase (EC 4.1.1.-) | -6.70 | 11.70 |
| *orf8222* | hypothetical protein | 2.43 | 2077.99 |
| *orf8289* | glycine-rich protein | 4.89 | 8.20 |
| *orf8372* | Amino acid/metabolite permease in hypothetical Actinobacterial gene cluster; BAT1-like | -3.56 | 10.44 |
| *orf8446* | [Protein-PII] uridylyltransferase (EC 2.7.7.59) | -4.23 | 1014.49 |
| *orf8447* | Nitrogen regulatory protein P-II | -4.47 | 1014.49 |
| *orf8448* | Ammonium transporter | -4.69 | 1014.49 |
| *orf8469* | Chitinase (EC 3.2.1.14) | -4.54 | 613.74 |
| *orf8886* | FIG00998525: hypothetical protein | 2.31 | 52.28 |
| *orf9120* | Phosphate regulon transcriptional regulatory protein PhoB (SphR) | -3.36 | 10.90 |
| *orf9121* | Phosphate regulon sensor protein PhoR (SphS) (EC 2.7.13.3) | -3.43 | 10.90 |
| *orf9122* | Phosphate transport system regulatory protein PhoU | -3.43 | 10.90 |
| *orf9219* | Phosphate ABC transporter, periplasmic phosphate-binding protein PstS (TC 3.A.1.7.1) | -7.16 | 203.59 |
| *orf9266* | Outer membrane stress sensor protease DegS | -5.61 | 42.16 |
| *orf9335* | L-proline glycine betaine binding ABC transporter protein ProX (TC 3.A.1.12.1) | -3.18 | 9.10 |
| *orf9414* | Oligopeptide ABC transporter, periplasmic oligopeptide-binding protein OppA (TC 3.A.1.5.1) | -2.77 | 3282.65 |
| *orf9470* | putative glycosyl hydrolase | 2.35 | 366.23 |
| *orf9598* | putative methyltransferase | 2.37 | 42.20 |

^a^2day-log_2_FC, from RNA-seq data, is log_2_(FPKM_Δ_*_mtrA_*/FPKM_WT_) collected after 48h fermentation;

^b^-log_10_Pvalue, from DAP-seq data, is an index indicating the reliability that MtrA binds to certain genes (the smaller the “Pvalue”, the bigger the “-log_10_Pvalue”, and the more likely MtrA is to bind to that gene).

**Table S6. Sequences of top10 binding regions in DAP-seq**

| **Regions** | **Rough sequences (<100 bp)** |
| --- | --- |
| *orf9414p* | gtttccgattggtcggcgcagcaggtcacggtcacatcacggcggggacacggaactattcgtgtgcggtcagtgaacttgac |
| *orf8222p* | gccgcggaacgggaccggggcgaggcggcgccgttatctgctcgttacctttggggaatgagcgacacg |
| *orf3285p* | ccgtcaccacacgtatatgcctccgccaaggttacggatcggtaacaaaacccaggttgttctcag |
| *orf8446p* | gcccgatgcgcgatctccggctgctcgattacgagcgccacaccctcgaatacgcgcttcgttgatcggccgtcaa |
| *orf3840p* | cgccgcgctgtgcgctccctatgccgccgctgtgtctagatgacggtcaggtatcgggctcgtgacggtcgttacacgggctttccccgcgaccggg |
| *orf8469p* | gaaatgtcacggaaatcgccacgctaagcaacaggacgctaccgtaccgtcgatggaagatcacgactgcataacagcagcttta |
| *orf9470p* | tgagccacgggtctctcccatgtaacaaactgaaacaactacggaacgtttctgttacacgccagtgaagcctgtcacggtgatggctgtcaa |
| *orf9219p* | gtgggacggaaatgtcggatatttaccggcgacagttatcgccacgagatgtgacggtgtcctgttcgtgactcctgcggctcgtg |
| *orf3212p* | tcctttggtactgattctgtgatcaaaacctgacccgagtgtgatccccgttaccacgacccggtcagcgaataatgcccgag |
| *orf1935p* | taaccttcgacgatccgtatttggatcaccacgagccgtcatggcaaaagttacgcgccggtcacaaatcggacaggcattcatgtgccaa |

**Table S7. Sequences of proven binding regions**

| **Regions** | **Rough sequences** |
| --- | --- |
| *A.de-orf459p* | cctgcatggtgacattccggacggtccgcggcttgtcggactgccgtgcacgtacgggcgaattgcgaatttgtgaccttcgggttaaggcttttcggcgatgtgagcgctggatcactcgccgggcggcagtccataatccggtgatataaatcagctgtgctgaccgttgatcacccgcgttacaagtgggtcgcgctg |
| *A.de-orf2614p* | gcctgcatcgtcgcacgtggccgcggtaacgctgccgttacatcaggcaacccaacgtttaccacgcagaaaccgtcgtcgatgcgtcctcgcgcaacctgtcaactgacaggtattccggtgcttcgaggaggcggtcttgtcc |
| *A.de-orf5652p* | gccttgggctgcttgaacgggctgacagcggtttcgtgaggggcgctcatgcgcatgctccatcgaagcaacgggtgattgataactacgttatacatcatcgcgcggaccgccgatcac |
| *A.de-orf9266p* | ccggaatcttccgatcgatctgaatagaagtcactacggtctatgacgtcgtgactaggccgtgacattcaaaactacgcatggaagccggaccatttccggcaggttaccggcgaaaacgcagaatgcgctcgcgcgcgccttatcgata |
| *S.co-redZp* | aagatcttcttgaggtggaaaccacttcgtatcagtctctcaccagggcctcccaaccccctctccaagtgtgcacacgcgtgctaagtttggccgcatgaggc |
| *S.co-cadRp* | gccccgaccagcggtgaaccctgctgacccggccgtaacgaagtcttcatgcccgtggc |
| *S.co-cpkADp* | tcaggcattccagcggcggcagttaccacgccgtgactgatcacctacccggtgtttct |
| *S.ve-cmlNF1p* | acactccttctccgcgccgggggtgtccaagtcgttagacacggcgttccgggcgttgc |
